# Therapeutic nucleus-access BNCT drug combined CD47-targeting gene editing in glioblastoma

**DOI:** 10.1186/s12951-022-01304-0

**Published:** 2022-03-04

**Authors:** Jiejian Chen, Qi Dai, QiYao Yang, Xiaoyan Bao, Yi Zhou, Haiqing Zhong, Linjie Wu, Tiantian Wang, Zhicheng Zhang, Yiying Lu, Zhentao Zhang, Mengting Lin, Min Han, Qichun Wei

**Affiliations:** 1 Institute of Pharmaceutics, Zhejiang Province Key Laboratory of Anti-Cancer Drug Research, School of Pharmaceutical Sciences, Zhejiang University, Hangzhou, 310058 China; 2 Department of Radiation Oncology, Key Laboratory of Cancer Prevention and Intervention, The Second Affiliated Hospital, College of Medicine, Zhejiang University, Hangzhou, 310058 China; 3 Department of Medical Oncology, Guangzhou First People’s Hospital, School of Medicine, South China University of Technology, Guangzhou, 510180 Guangdong China

**Keywords:** Boron neutron capture therapy, Boron agent, Nanoliposome, Nucleus-targeting, Glioma, CD47

## Abstract

Glioblastoma is the most common brain primary malignant tumor with the highest mortality. Boron neutron capture therapy (BNCT) can efficiently kill cancer cells on the cellular scale, with high accuracy, short course and low side-effects, which is regarded as the most promising therapy for malignant brain tumors like glioma. As the keypoint of BNCT, all boron delivery agents currently in clinical use are beset by insufficient tumor uptake, especially in the tumor nucleus, which limits the clinical application of BNCT. In this study, nuclear targeting of boron is achieved by DOX-CB, consisting of doxorubicin (DOX) and carborane (CB) utilizing the nuclear translocation property of DOX. The nucleus of GL261 cells takes up almost three times the concentration of boron required for BNCT. To further kill glioma and inhibit recurrence, a new multifunctional nanoliposome delivery system DOX-CB@lipo-pDNA-iRGD is constructed. It combines DOX-CB with immunotherapy strategy of blocking macrophage immune checkpoint pathway CD47-SIRPα by CRISPR-Cas9 system, coupling BNCT with immunotherapy simultaneously. Compared with clinical drug Borocaptate Sodium (BSH), DOX-CB@lipo-pDNA-iRGD significantly enhances the survival rate of tumor-bearing mice, reduces tumor stemness, and improves the prognosis. The excellent curative effect of this nanoliposome delivery system provides an insight into the combined treatment of BNCT.

**Graphical Abstract:**

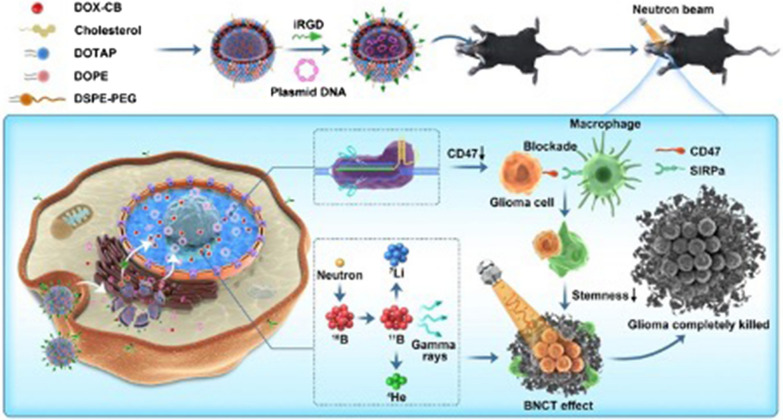

**Supplementary Information:**

The online version contains supplementary material available at 10.1186/s12951-022-01304-0.

## Introduction

Glioblastoma is the most common primary malignant tumor in the central nervous system. The median survival time of newly diagnosed gliomas is 8–15 months, respectively [1–3]. Recurrence and metastasis are the main causes of treatment failure and lethality of malignant glioma. Almost all malignant gliomas relapse after treatment [4], however, there is a lack of effective treatment for recurrent glioma. Thus, it is imperative to explore new treatment strategies.

BNCT is a binary biological targeted radiotherapy technology based on nuclear capture and fission reaction [5]. The stable nonradioactive isotope Boron 10 (^10^B) reacts with low-energy thermal neutrons to induce nuclear decay and fission, resulting in high-energy α particle (^4^He) and recoil lithium core (^7^Li). BNCT combines the advantages of biological target and heavy ion radiotherapy. It achieves accurate and efficient attack on tumor cells through generating an obvious boron concentration gradient between tumor cells and normal cells, thereby minimizing the toxic and side effects on normal tissues [6, 7]. BNCT is considered to be a promising tumor treatment method.

According to the clinical study of Studisvk in Sweden, the median survival time of glioblastoma treated with BNCT alone after operation is 14.2 months [6]. The median survival time of glioblastoma treated with BNCT followed by conventional radiotherapy of 20–30 Gy after BNCT treatment in Osaka Medical College in Japan is 23.5 months [8]. The results are encouraging. However, at the current stage, two clinical drugs used in BNCT, BSH and Boronophenylalanine (BPA), have the shortcomings of low targeting and boron-containing. Although with high injection dose of BPA and BSH, the ratio of boron concentration in tumor tissue to that in surrounding normal tissue or blood still fails to reach the ideal ratio [9, 10]. Therefore, it is necessary to construct a nanoparticle for enhanced tumor targeting and reduces the tumor recurrence rate.

From the aspect of the therapeutic mechanism of BNCT, because the range of ^4^He and ^7^Li produced by BNCT is very short, which is only 5–9 μm, the intracellular localization of ^10^B directly affects the therapeutic effect. The closer to the tumor nucleus, the higher the probability of the high linear energy transfer rays produced by fission of ^10^B hitting the DNA in the nucleus, and the easier to kill the tumor cells. Localization of ^10^B in the nucleus is ten times more effective than that in the cytoplasm [11, 12]. Therefore, we choose DOX, which has an excellent nuclear tropism, as the carrier for boron delivery. The intracellular transport pathway of DOX is closely related to proteasome action [13, 14], the latter entering the nucleus through the NLS structure [15]. Based on this characteristic of DOX, DOX-CB was constructed and is assumed to deliver boron capture agents into the nucleus through the interaction between proteasome and DOX.

Considering of reducing recurrence rate, CD47, the Integrin-Associated protein, is chosen to be the target of combined immunotherapy. It can be regarded as the “Don’t Eat Me” signal by binding its receptor SIRP α (signal regulatory protein α) on phagocytic cells to inhibit signal transmission and prevent phagocytosis of tumor cells [16, 17]. CD47 blocking immunotherapy can effectively activate macrophage-mediated phagocytosis, promote adaptive immunity, and reduce the risk of recurrence [18]. Current studies have revealed that monoclonal antibodies targeting CD47 and SIRPα show therapeutic effect in preclinical models of many solid tumors and hematological tumors by promoting macrophage phagocytosis on cancer cells and cancer stem cells, as a new way of tumor immunotherapy [19, 20]. CRISPR-Cas9 is a powerful gene editing technology that provides a convenient and effective tool for immunity research by using a single gRNA-directed Cas9 nuclease [21–24]. Utilizing this system to directly knock out the CD47 gene can not only promote the phagocytosis of macrophages but also increase the efficacy of BNCT treatment, so as to reduce the recurrence rate of tumor from two aspects.

In order to increase the tumor targeting of nanoparticles, we selected the Internalizing-RGD (iRGD), which is a homing peptide with specific integrin targeting and satisfied tumor penetration and accumulation [25, 26]. In brief, the iRGD peptide binds to intern αv β3/5 expressing tumor cells (breast cancer, gliomas, pancreatic tumor and so on) and tumor blood vessel endothelial cells, and then cleaved by proteases to expose the CendR component. The CendR could conjugate to neuropilin-1 and penetrates into cells and tissue. The peptide could still keep good penetration into tumors even grafting cargo attached to the N-terminus of the iRGD.

**Fig. 1:**
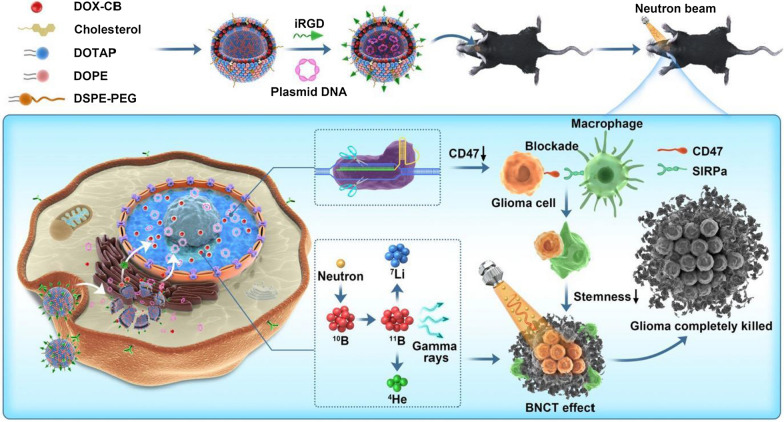
Schematic illustration of therapeutic mechanism of DOX-CB@lipo-pDNA-iRGD

Herein, we established a multifunctional nanoliposome delivery system, DOX-CB@lipo-pDNA-iRGD, based on the tumor targeted delivery ability of iRGD and the nuclear tropism of DOX, to deliver the CD47 gene-targeted CRISPR-Cas9 gene knock-out plasmid and boron delivery drug to the nucleus of tumor tissue as much as possible **(**Fig. 1). After the plasmid enters the nucleus, it is transcribed and translated into Cas9 protein/sgRNA complex to play the role of gene editing. Using the BNCT effect of DOX-CB combined with the improved phagocytosis of CD47-blocked tumor cells and tumor stem cells, the study increased anti-tumor effectiveness, reduced the recurrence rate, and prolonged the survival time.

## Results and discussion

### Gene transfection efficiency of the pDNA-lipoplexes (lipo-pDNA)

**Fig. 2:**
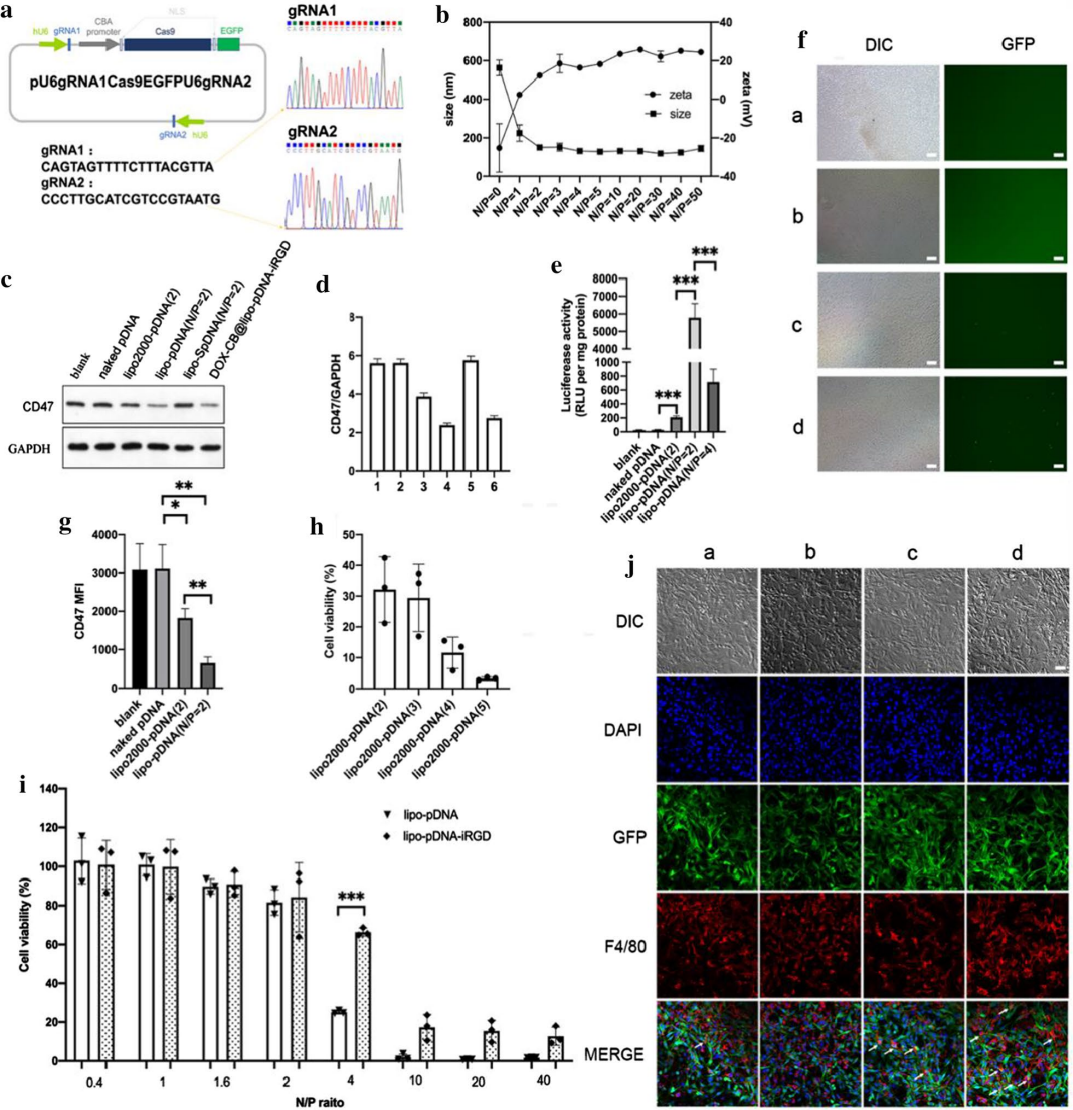
Characterization of pDNA-lipoplexes (lipo-pDNA) and the functional validation of pDNA-lipoplexes (lipo-pDNA) in vitro. **a** Schematic diagram of pU6gRNA1Cas9EGFPU6gRNA2 plasmid and sgRNAs targeting CD47 gene. **b** The change of particle sizes and zeta potentials of pDNA-lipoplexes (lipo-pDNA) at various N/P ratio. **c,** Expressions of CD47 in GL261 cells transfected with different preparations. Lipo-SpDNA is short for liposome-scrambled pDNA. **d,** Western blot-based quantifications of CD47 expressions in GL261 cells transfected with different preparations. The results are expressed as mean ± SD (n = 3). **e,** CD47 expressions on cells were analysed by Firefly Luciferase Reporter Gene Assay Kit after GL261 cells were transfered with different preparations for 24 h. Data are expressed as mean ± SD (n = 3). ***P < 0.001. **f** Fluorescence picture GL261 cells after transfection of 48 h with (a) blank, (b) PEI-pDNA, (c) lipo2000-pDNA (2), (d) lipo-pDNA (N/P = 2) (2.5 μg/mL equivalent pDNA). Scale bar = 100 μm. **g** CD47 expressions on cells were analysed by flow cytometry after GL261 cells were transfered with different formations for 72 h. Data are expressed as mean ± SD (n = 3). *P < 0.05, **P < 0.01. **h** Evaluation of cell viability (CCK-8 assay) of GL261 cell lines after 24 h of treatment with different preparations, respectively. Values are expressed as mean ± SD (n = 6). **i** Evaluation of cell viability (CCK-8 assay) of GL261 cells after 24 h of treatment with different preparations, respectively. Values are expressed as mean ± SD (n = 6). **j** Confocal fluorescentmicroscopy of macrophages 8 h after phagocytosis of GL261 cells precultured with (A) blank, (B) naked pDNA, (C) lipo2000-pDNA (2) and (D) lipo-pDNA (N/P = 2) for 72 h. Scale bar = 60 μm. The green fluorescence is for GL261 tumor cells and the red fluorescence is for macrophages

The key to the preparation of multifunctional nanocomposites is the successful construction of cationic liposome-plasmid complexes. Two sgRNA sequences were designed on the plasmid to knock out the CD47 gene of tumor cells (Fig. 2a) [27, 28]. Based on the principle of charge interactions, cationic liposome-plasmid complex can be prepared by the mixed incubation of cationic liposome and plasmid. Firstly, the particle size and surface potential of lipo-pDNA with different N/P ratios were measured (Fig. 2b and Additional file 1: Table S1). As an indicator representing the ratio of cationic liposome to plasmid, the N/P ratio has a significant effect on the particle size, dispersion and surface potential of the pDNA-lipoplexes (lipo-pDNA), which directly determines the final transfection efficiency of the complex. The pure plasmid solution, with an N/P ratio of 0, showed − 25.25 ± 12.63 mV potential and 562.97 ± 40.24 nm particle size, indicating the agglomeration of exposed plasmids. Particles with N/P ratios ranging from 1 to 2 showed reduced particle sizes from 225.25 ± 43.21 nm to less than 150 nm and positive surface potential. According to the agarose gel retardation assay, pDNA was effectively compressed and protected by the liposome in cases of N/P ratios over 1.6, especially when N/P was 2 (Additional file 1: Fig. S1). Hence, the complex with an N/P ratio of 2 was chosen for subsequent experiments.

To demonstrate the transfection efficacy, Western blot analysis was used to examine the CD47 expression in GL261 cells. As Fig. 2c indicates, all the GL261 cells transfected with different preparations exhibited moderate CD47 expressions, which, however, decreased significantly in pDNA-lipoplexes transfection groups. The quantitative analysis of protein expressions conducted by Image J software further revealed intuitively the superiority of our liposome over the commercial lipofectamine 2000 (Fig. 2d). The transfection efficiency of pDNA-lipoplexes was further determined through the transfection of pGL3 and green fluorescent protein (GFP). The experimental group lipo-pDNA (N/P = 2) showed the highest gene transfection efficiency according to the GFP signals (Fig. 2e and f). In comparison, lipo2000-pDNA (2) and PEI-pDNA, serving as two positive control groups, exhibited poor transfection effects. These results demonstrated higher gene transfection capacity of the proposed nanoliposomes than the commercial products lipo2000 and PEI.

### Functional validation of pDNA-lipoplexes (lipo-pDNA) in vitro

The ideal outcome after transfection of CRISPR-Cas9 knock-out plasmid targeting CD47 gene is to reduce the expression level of CD47. In the experiment lipo2000 was used as the control, and the expression level of CD47 after transfection was detected by flow cytometry. As shown in Fig. 2g, the expression level of CD47 was similar between the plasmid group and the blank control group. The expression level of CD47 in lipo2000-pDNA (2) group was obviously decreased. While the expression of CD47 in lipo-pDNA (N/P = 2) group was lower than that in lipo2000-pDNA (2), with a significant difference discovered between the two groups (P < 0.01). The expression of CD47 in lipo-pDNA (N/P = 4) group was also lower than that in lipo2000-pDNA (2) (P < 0.01), which further indicated the superior transfection effect of the nanoliposomes and provided the possibility to achieve immune activation through drug administration in vivo.

The cytotoxicity of lipo-pDNA and lipo-pDNA-iRGD in different N/P ratios against GL261 cells for 24 h was examined by CCK8 assay, and the results are featured in Fig. 2i. When the N/P ratio was less than or equal to 1, lipo-pDNA and lipo-pDNA-iRGD showed no toxic effect on GL261 cells. T Survival rates of cells transfected by complexes with an N/P ration of 2 was also higher than 50%. With an N/P ratio of 4, the cytotoxicity of the complex was significantly increased, and the cell survival rate was less than 25%. As we mentioned in Fig. 2b, it may enhance the cell cytotoxicity because of the increase of the positivity charge of the lipo-pDNA. And under the same N/P ratio, a neutral surface potential of the lipo-pDNA-iRGD tended to elicit relatively low cytotoxicity. In all of the different ratios, the cytotoxicity of the lipo2000-pDNA were greater than lipo-pDNA, which reflects the safety advantages of our multifunctional nanoliposomes (Fig. 2h).

As mentioned above, the ideal outcome after transfection is to reduce the expression level of CD47 in tumor cells, and thus restore or promote the phagocytosis of macrophages. In this study, primary bone marrow stem cells from C57BL/6 mice were extracted and stimulated to differentiate into macrophages for using in the cell phagocytic assay. As the Fig. 2j showed, in the plasmid group, tumor cells were rarely phagocytized; in the blank control group, only one tumor cell was engulfed by macrophages. As for lipo2000-pDNA group, a degree of macrophage phagocytosis could be observed under microscope. The phagocytosis was more obvious in GL261 cells transfected with lipo-pDNA (N/P = 2) than those with lipo2000-pDNA, which indicated that the expression level of CD47 gene in tumor cells decreased after lipofectamine transfection, thus improving the phagocytosis of macrophages. The nanoliposomes constructed by us hold a good application prospect in promoting phagocytosis.

### Characterization and in vitro experiments of DOX-CB

To establish an efficient drug delivery platform for combination therapy of BNCT and immunotherapy, we designed a multifunctional nanoliposome, which carries the boron-containing drug DOX-CB and the CRISPR-Cas9 plasmid for CD47 gene knock-out. Before the preparation of DOX-CB@lipo-pDNA-iRGD, the structure of DOX-CB needs to be confirmed first. According to the analysis of ^1^H-NMR, the DOX-CB not only retains the chemical shifts of several groups corresponding to DOX structure, but also has an obvious absorption peak at the shift of 1.5 ~ 3.5 ppm, which is consistent with the absorption peak of B-H group in CB (Additional file 1: Fig. S2) [29, 30]. This preliminarily indicates that DOX-CB does contain DOX and CB. They may interact with each other through π–hydrogen bond, hydrogen bond, or dihydrogen bond to form complex internal structures. Employing two-dimensional NOESY spectra, we found that the benzene ring in the DOX may interact with the B–H group in CB (indicated by the two black frames), and the B–H group (indicated by the black arrow) in the boron cage may interact with the C–H, N–H and other groups in the DOX structure, which form noncovalent bonds such as dihydrogen bonds (Additional file 1: Fig. S3) [31, 32]. These forces can promote the relative stability of the composite structure. There are a series of absorption peaks in the ^11^B-NMR spectra of CB but no in DOX because it has no boron molecular structure (Additional file 1: Fig. S4). The absorption peak of DOX-CB can be observed, which shows that DOX-CB is a boron-containing complex. The complex intermolecular interaction exists in the internal structure of DOX-CB complex, which may change the chemical shift of ^11^B-NMR spectra assignment. In the IR spectra of CB, B-H showed an obvious typical vibration peak at 2593 cm^−1^, and DOX has no obvious bending or stretching vibration peak near this wavelength (Additional file 1: Fig. S5). The absorption peak near the wavelength of 2593 cm^−1^ also appears in the infrared spectrum of DOX-CB, which indicates that DOX-CB contains a boron cage, and boron is an indispensable component of boron neutron capture therapy. After measurement by ICP-MS three times, the content of boron in DOX-CB is 4.79% ± 0.16% (Additional file 1: Fig. S6). The above experimental results confirm that DOX-CB is a complex composed of DOX and CB through a variety of intermolecular forces, but it is unknown whether the formation of a new spatial structure will affect the fluorescence characteristics of DOX. Herein, we detected the UV absorption peak of DOX, CB and DOX-CB. As shown in Additional file 1: Fig. S7, DOX has an obvious absorption peak at 480 nm, while CB has no absorption peak in the whole range of experimental wavelength. 480 nm is taken as the maximum absorption wavelength of DOX. The UV absorption spectrum of DOX and CB after simple physical mixing is almost the same as that of DOX. After a series of preparation process, the maximum absorption peak (480 nm wavelength) of DOX-CB at the same molar concentration decreased significantly, which may be due to the molecular interaction within DOX-CB that reduced the fluorescence absorption capacity of DOX. After mixing DOX-CB with triethylamine (TEA) to remove the ionized state, the UV absorption spectrum was basically unchanged, indicating that the structure of DOX-CB was relatively stable. As Additional file 1: Fig. S8A indicated, the amino groups on DOX (C27H31NO11, MW = 545.53) reacted with bromine on 1-Bromomethyl-*o*-carborane (C3H2B10Br, MW = 226.06) to form DOX-CB (C30H32NO11B10, MW = 690.68) and hydrogen bromide. The characteristic peak of 1-Bromomethyl-o-carborane was about 218 (Additional file 1: Fig. S8B) and DOX was about 544 (Additional file 1: Fig. S8C). The characteristic peak of DOX-CB was about 683 (Additional file 1: Fig. S8D), and there had not the characteristic peak in 544 and 218, which illustrated that there is neither free DOX nor CB in DOX-CB. And the spectrum of DOX-CB nanoparticle (Additional file 1: Fig. S8E) also did not have the characteristic peak in 544 and 218, but had the characteristic peak in 683, which further confirmed that the drug contained in the preparation was DOX-CB.

As for in vitro cytotoxicity assay, DOX-CB shows a concentration dependent cytotoxicity on GL261 cells, but its cytotoxicity is much smaller than DOX group, with an IC50 value exceeding 10 μg/mL (Additional file 1: Fig. S9). On the one hand, DOX-CB may contain the main chemical structure of DOX, which leads to a certain degree of cytotoxicity; on the other hand, due to the complex internal structure of DOX-CB, the cytotoxic effect of DOX-CB cannot be exactly the same as that of DOX, and the existence of CB inevitably leads to the change of the cytotoxic effect of DOX-CB. The survival rates of other tumor cell lines (U87 and C6 glioma cells) after incubation with DOX for 48 h were lower than 20%, which indicated that these two kinds of glioma cells, like GL261 cells, were sensitive to DOX. The cytotoxicity of DOX-CB was also weaker than DOX (Additional file 1: Fig. S10).

**Fig. 3:**
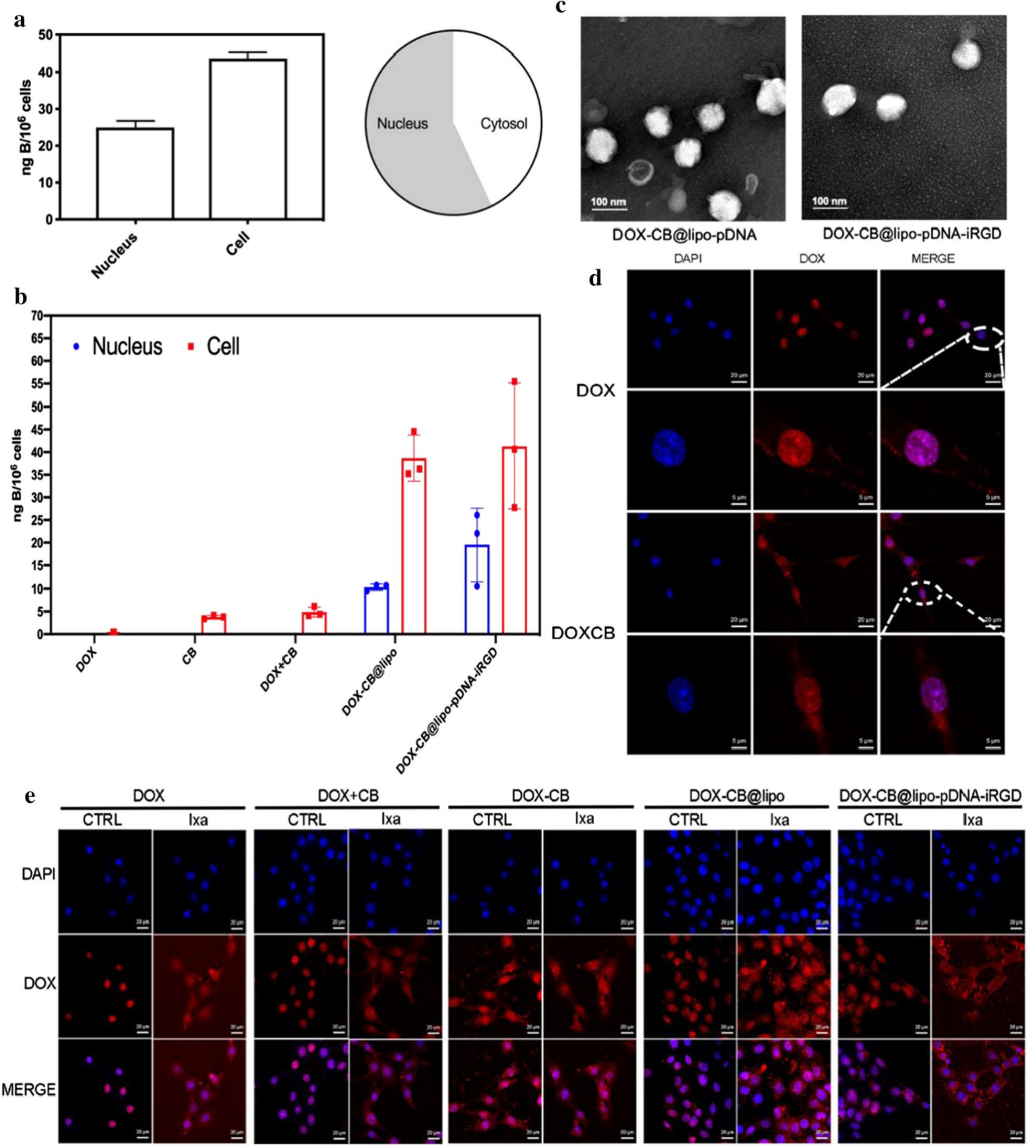
Characterization and in vitro experiments of DOX-CB@lipo-pDNA-iRGD. **a** Measurement of intranuclear and cellular boron uptake in GL261 cells. Cells were incubated for 3 h in the presence of DOX-CB at the dose of 16 μg/mL. **b** Measurement of intranuclear and cellular boron uptake in GL261 cells. Cells were incubated for 3 h in the different preparations at the dose of 16 μg/mL. **c** Transmission electron microscopic (TEM) image of DOX-CB@lipo-pDNA (B) and DOX-CB@lipo-pDNA-iRGD. Scale bar = 100 nm. **d** Confocal microscopy showing the cellular distribution of DOX and DOX-CB. GL261 cells were incubated with DOX or DOX-CB at the equivalent DOX concentration of 3.5 μM for 3 h. **e** Confocal microscopy showing the cellular distribution of different formations with or without proteasome inhibitor

In order to determine the spatial distribution and fluorescence intensity of DOX-CB in cells, three cell lines have been observed. As shown in Fig. 3e, the red fluorescence corresponding to free DOX was mainly located in the nucleus of GL261, while the red fluorescence corresponding to DOX-CB was mainly located in the cytoplasm and mostly in the nucleus. In the experiment of U87 cells, the DOX-CB fluorescence was partly located in the cytoplasm and the nucleus respectively (Additional file 1: Fig. S11). In another cell line C6, the fluorescence corresponding to free DOX was basically located in the nucleus, while a small part of DOX-CB was located mostly in the nucleus rather than the cytoplasm (Additional file 1: Fig. S12). The results showed that in DOX-CB group, the fluorescence in the nucleus was stronger than that in the cytoplasm. It is also found that the excitation intensity of DOX-CB group is slightly weaker than that of DOX group, which is consistent with the result in Additional file 1: Fig. S7 showing that the interaction between CB and DOX affects the fluorescence characteristics of DOX-CB.

### Cellular uptake and distribution of DOX-CB@lipo-pDNA-iRGD

The results of the cell fluorescence distribution experiment showed that the preparation had a nuclear tendency. The application of BNCT in the treatment of tumors requires that the selective uptake of boron by tumor cells should be at least 20 μg boron per gram of tumor tissue, equivalent to 10^9^ boron atoms per cell (about 16.67 ng B/10^6^ cells). Therefore we further determined the boron concentration in the cell especially in nucleus by ICP-MS, calculated the ratio of boron content in the cell nucleus and cytoplasm, and analyzed the tendency of the preparations in the intracellular substructure. As shown in Fig. 3c, after incubation with DOX-CB for 3 h, the boron uptake of the nucleus of GL261 cells was 24.96 ± 1.81 ng/10^6^ cells, while the boron uptake of GL261 cells was 43.71 ± 1.51 ng/10^6^ cells. The graph showed that the boron content in the nucleus was higher than that in the cytoplasm. Compared to the DOX and CB mixture group which had no boron uptake in nucleus, it illustrated that the chemical bonding of DOX and CB is the key factor for DOX to carry CB into the nucleus. In the experiment, CB was used as the control, and the boron uptake of whole cells was only 3.79 ± 0.53 ng/10^6^ cells, with no boron uptake in nucleus (Fig. 3d). This shows that CB itself has limited ability to enter the nucleus, and DOX-CB complex effectively promotes the nucleus entrance of CB. The boron uptake by the nucleus of GL261 cells in DOX-CB@lipo group and DOX-CB@lipo-pDNA-iRGD group were 10.37 ± 0.71 ng/10^6^ cells and 19.56 ± 8.05 ng/10^6^ cells, while the boron uptake by the whole GL261 cells were 38.68 ± 5.11 ng/10^6^ cells and 41.28 ± 13.87 ng/10^6^ cells. The boron uptake of DOX-CB@lipo-pDNA-iRGD by GL261 tumor cells measured by ICP-MS is at an ideal level. In particular, DOX can carry boron into the nucleus after entering the cells, which will greatly improve the therapeutic effect of BNCT. The DOX-CB@lipo-pDNA-iRGD exhibit superior nuclear tendency according to the above results, proving potential as a nuclear targeted boron capture agent with good application prospect of BNCT.

At present, it is generally considered that DOX enters the nucleus with the assistance of proteasome and embeds DNA in the nucleus to affect cell proliferation, so as to achieve the antitumor effect. To confirm the therapeutic pathway of our nanoliposome, we investigated the cellular distribution of different formations with or without proteasome inhibitors. From Fig. 3f we could confirm that, compared to the groups dealing without proteasome inhibitor, most of DOX in the formations dealing with proteasome inhibitor were distributed in the periphery of the nucleus, especially the DOX-CB@lipo group and DOX-CB@lipo-pDNA-iRGD group, indicating that the intracellular transport pathway of our nanoliposome is related to proteasome action.

### Anti-tumor effect of BNCT combined immunochemotherapy in vivo

We successfully established the mice model of glioma in situ by stereotaxically injecting GL261 cells into the left brain of C57BL/6 male mice aged 5–6 weeks. The examination of MRI (Additional file 1: Fig. S13a–c) and in vivo bioluminescent imaging (Additional file 1: Fig. S13d) showed that GL261 tumor tissue was growing in the left hemisphere of the mouse brain, and the modeling was successful. Also, the result of HE staining in Additional file 1: Fig. S14 indicated that the blood–brain barrier would be damaged during the development of brain tumor in tumor bearing mice, which was similar to the actual clinical phenomenon. To investigate the ability of the nanoliposomes to deliver drugs into the brain tumor region, the cationic liposomes physically encapsulated with DiR (DiR@lipo) and DiR@lipo-iRGD were prepared by mixing the peptide iRGD with DiR@lipo, and the distribution of these two drugs was observed by fluorescence imaging in vivo after administration (Additional file 1: Fig. S15a–d). The fluorescence distribution of DiR@lipo-iRGD experimental group in the brain region was obvious, which indicated a high drug delivery efficiency. After animal dissecting, the results also showed a superior drug delivery ability of DiR@lipo-iRGD in brain tumors (Additional file 1: Fig. S16a–e).

**Fig. 4:**
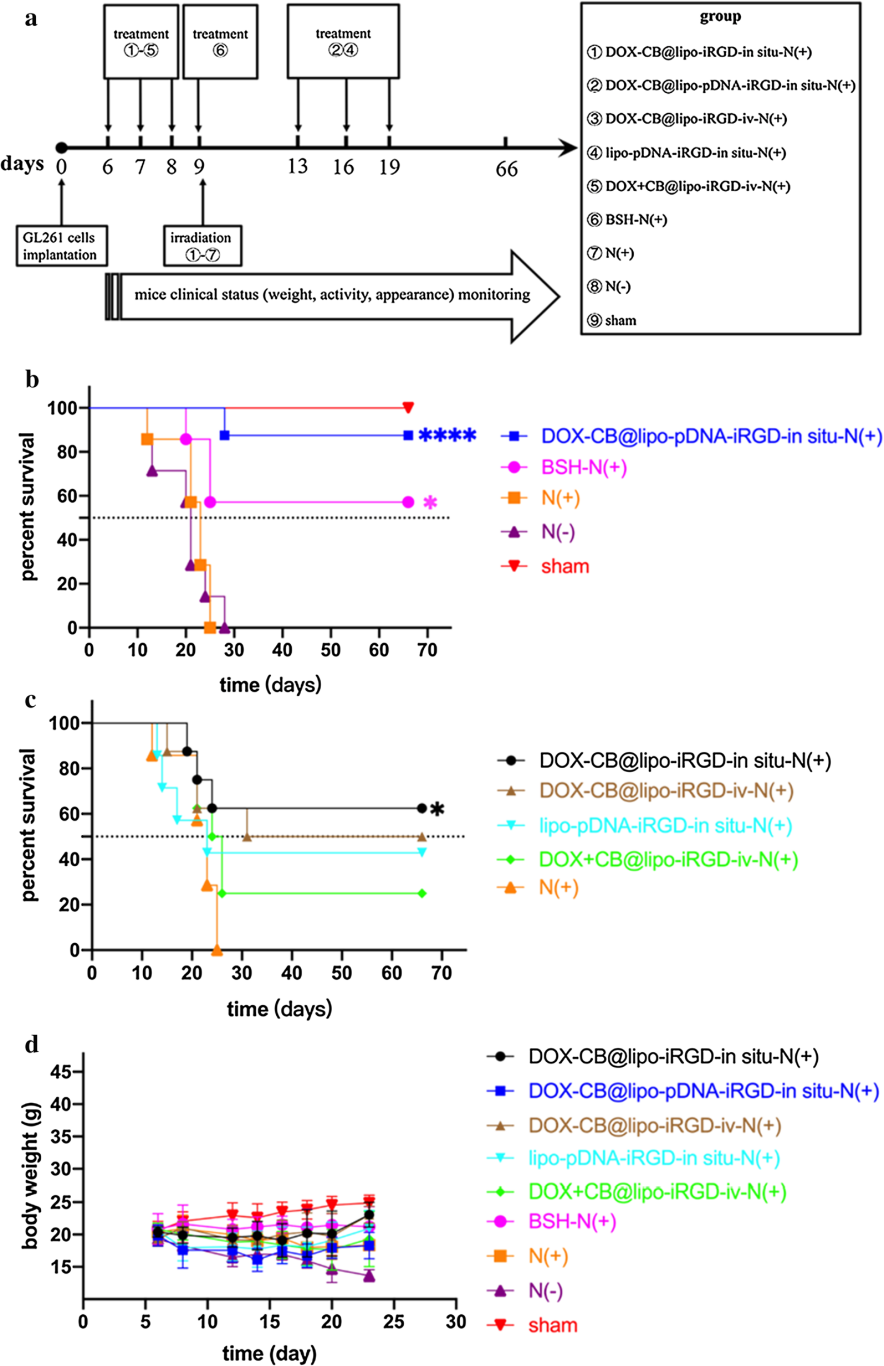
Anti-tumor effect of BNCT combined immunochemotherapy in vivo. **a** Schematic illustration of treatment procedure. **b**, **c** Kaplan–Meier survival curve and **d** body weight curve of Gl261 tumor-bearing mice with different treatments. Log-rank (mantel-cox) test was used for statistical analysis of **b** and **c**. ****P < 0.0001 vs group N(+) in **b**; *P = 0.0160 vs group N(+) in **b**; *P = 0.0312 vs group N( +) in **c**

The anti-tumor experiment is schematically illustrated in Fig. 4a. There were nine groups including the DOX-CB@lipo-iRGD-in situ-N (+) group, the DOX-CB@lipo-pDNA-iRGD-in situ-N (+) group, the DOX-CB@lipo-iRGD-iv-N (+) group, the lipo-pDNA-iRGD-in situ-N (+) group, the DOX + CB@lipo-iRGD-iv-N (+) group, the BSH-N (+), the N (+) group, the N (−) group, and the Sham group. Thermal neutron is a relatively safe radiation, which has no obvious side effect on the survival of mice. As shown in Fig. 4b, tumor-bearing mice in thermal neutron irradiation group (group 7) and blank control group (group 8) died within 30 days, indicating that GL261 glioma in situ progressed rapidly without treatment and was life-threatening, which well simulated the dangerous biological characteristics of clinical glioma. In the experimental group 9, the mice survived well and showed relatively higher average body weights (Fig. 4d), indicating that the experimental operation did not cause obvious acute postoperative complications in mice, and the interference of operation factors on the experiment was excluded.

The survival rate of tumor bearing mice in experimental group 3 was significantly higher than that in experimental group 5, which may be related to the nuclear tropism of boron delivering agent. In experiment group 5, DOX and CB reagents were encapsulated separately, and this agent was not proved to have nuclear tropism. While in experiment group 3, DOX-CB complex was encapsulated with nuclear tropism and exhibited more significant therapeutic effect of BNCT. However, there was no significant difference in the survival rates of the tumor bearing mice in the experimental groups 1 and 3, which was basically the same as that in the BSH (group 6). On the one hand, due to the low efficiency of intravenous injection, the bioavailability of brain targeted drug delivery is less than 10% ID/g. Even though the dose of the intravenous injection group was about one order of magnitude higher than that of the intratumoral injection group, the concentration of the preparation reaching the brain tumor target area was equivalent to the intratumoral injection method, resulting in the same final curative effect. On the other hand, BSH is one of the two boron drugs in clinical application of BNCT, with a very high clinical boron dose of 100 mg/kg. However, the dose of our multifunctional nano liposomes in this experiment is far lower. After 66 days of observation, it was found that there was no significant difference in the survival rate of mice. These results indicate that multifunctional nanoliposomes with high-efficiency nuclear drug delivery can enhance the therapeutic effect to an extent.

Experimental group 2 was treated by intratumoral administration of boron neutron capture immuno-chemotherapy. After treatment, the survival rate of tumor bearing mice was very high, and the curative effect was clear. Only one mouse died at the 66th day, and it died within 30 days after modeling, which could not rule out the failure caused by drug leakage in the process of administration. A range of literatures [33, 34] have pointed out that blocking CD47 driven macrophage-mediated immunotherapy has a definite curative effect, but it is recommended to be combined with other therapies. In this experiment, the survival rate of mice treated with immunotherapy alone (experimental group 4) was less than 50%, which may be related to the limited ability to block CD47 on immune activation. It should be pointed that although the multi-functional nanoliposomes constructed by us could deliver the CRISPR/Cas9 gene knock-out plasmid of CD47 gene to tumor cells, we could not avoid its off-target effect. However, due to the lack of the "eat me" signals unique to tumor cells in normal tissue cells, it is relatively difficult to trigger a strong immune response even if the "don't eat me" signal of normal tissue cells is reduced due to the off-target effect of some multifunctional liposomes [35]. As shown in Fig. 4d, the weight of tumor bearing mice in each group was basically within the normal range after treatment, showing certain safety of the preparations.

### In vivo immunological evaluation

**Fig. 5:**
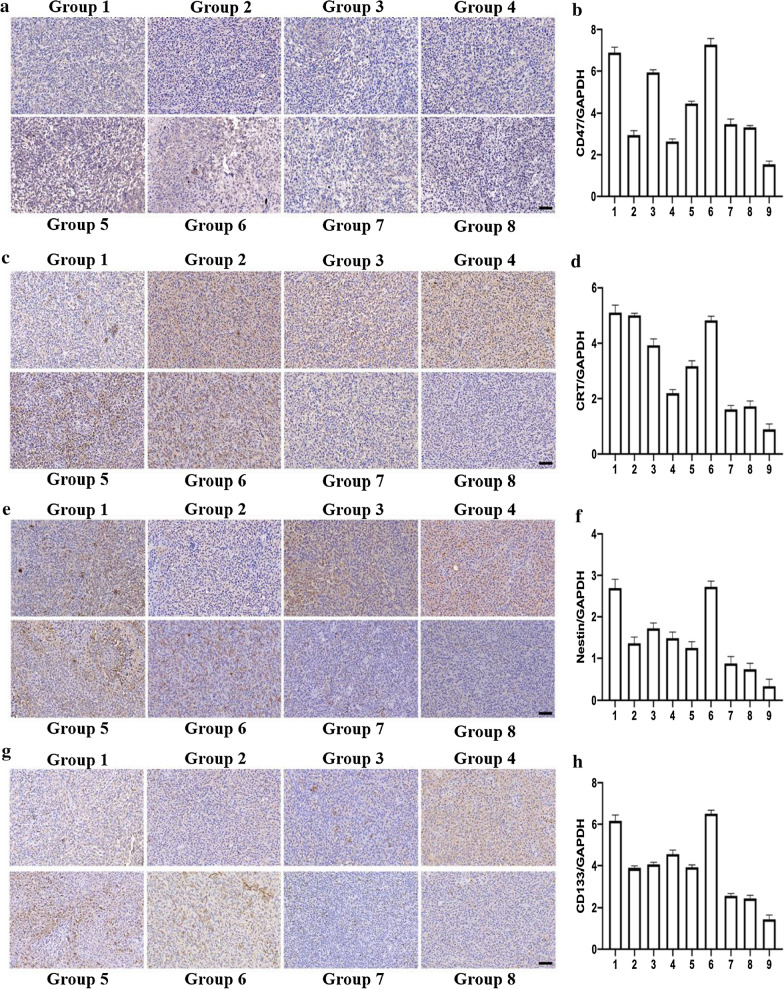
Western Blot and immunohistochemical assay. **a** CD47 immunohistochemical staining in gliomas of tumor bearing mice in group 1–8. **b** Western blot-based quantifications of expression of the CD47 in group 1–9. **c** CRT immunohistochemical staining in gliomas of tumor bearing mice in group 1–8. **d** Western blot-based quantifications of expression of the CRT in group 1–9. **e** Nestin immunohistochemical staining in gliomas of tumor bearing mice in group 1–8. **f** Western blot-based quantifications of expression of the Nestin in group 1–9. **g** CD133 immunohistochemical staining in gliomas of tumor bearing mice in group 1–8. **h** Western blot-based quantifications of expression of the CD133 in group 1–9. The results of western-blot are expressed as mean ± SD (n = 3). Group 1–9 correspond to the DOX-CB@lipo-iRGD-in situ-N (+) group, the DOX-CB@lipo-pDNA-iRGD-in situ-N (+) group, the DOX-CB@lipo-iRGD-iv-N (+) group, the lipo-pDNA-iRGD-in situ-N (+) group, the DOX + CB@lipo-iRGD-iv-N (+) group, the BSH-N (+), the N (+) group, the N (−) group, and the Sham group. Group 1–8 correspond to the DOX-CB@lipo-iRGD-in situ-N (+) group, the DOX-CB@lipo-pDNA-iRGD-in situ-N (+) group, the DOX-CB@lipo-iRGD-iv-N (+) group, the lipo-pDNA-iRGD-in situ-N (+) group, the DOX + CB@lipo-iRGD-iv-N (+) group, the BSH-N (+), the N (+) group, and the N (−) group

The immunological mechanism was further verifie by Western blot (Additional file 1: Fig. S17). The CD47 gene expression of the second and the fourth groups significantly decreased compared with the first, the third and the fifth groups (Fig. 5b), showing the high efficiency of the CRISPR-Cas9 system. CD47 gene is closely related to cancer stem cells. Overexpressed CD47 is seen in ovarian cancer [36], brain tumors [37, 38] lung tumors [39, 40] and other tumors [41–44], especially in cancer stem cells, and the high expression of CD47 is closely related to tumor growth, differentiation, proliferation, metastasis and recurrence, causing the poor prognosis in patients. Therefore, the down-regulation of CD47 gene expression may indicate the decrease of cell stem, which is more conducive to macrophages to phagocytize residual stem cells.

We also detected other glioma stem cell (GSC) markers, such as CRT (Fig. 5d), Nestin (Fig. 5f), CD133 (Fig. 5h) and CD15 (Additional file 1: Fig. S18). The results showed that Nestin and CD133 markers were decreased in the second to the fifth treatment groups, indicating that after our immune and BNCT combination treatment, the stemness of GSC is reduced. The expression of CD133 and Nestin is deeply involved in the occurrence, invasion, tumor differentiation, infiltration, and the degree of malignancy [45–48]. Therefore the decreased CD133 and Nestin expression may affect the ability of tumor self-renewal and multidirectional differentiation and thus reduce the recurrence rate and improve the prognosis. CRT is a non-specific tumor stem index, and the effect of CD15 on GSC is not clear [49, 50], which may explain their slight expression decline. It is worth noting that in the sixth group, which using the clinical BNCT drug BSH, all the GSC markers were not decreased, contrary to the second to the fifth treatment groups. This may be the reason why there is still recurrence after BNCT treatment in the current stage. It also reveals that our nanoliposome system may be better than BSH in preventing tumor recurrence.

In the examination of liver function, it shows that the second group, DOX-CB@lipo-pDNA-iRGD-in situ group, has no significant effect on the liver injury compared with the control group, and is slightly less than that in the BSH group (Additional file 1: Fig. S19).

### Anti-tumor evaluation

**Fig. 6:**
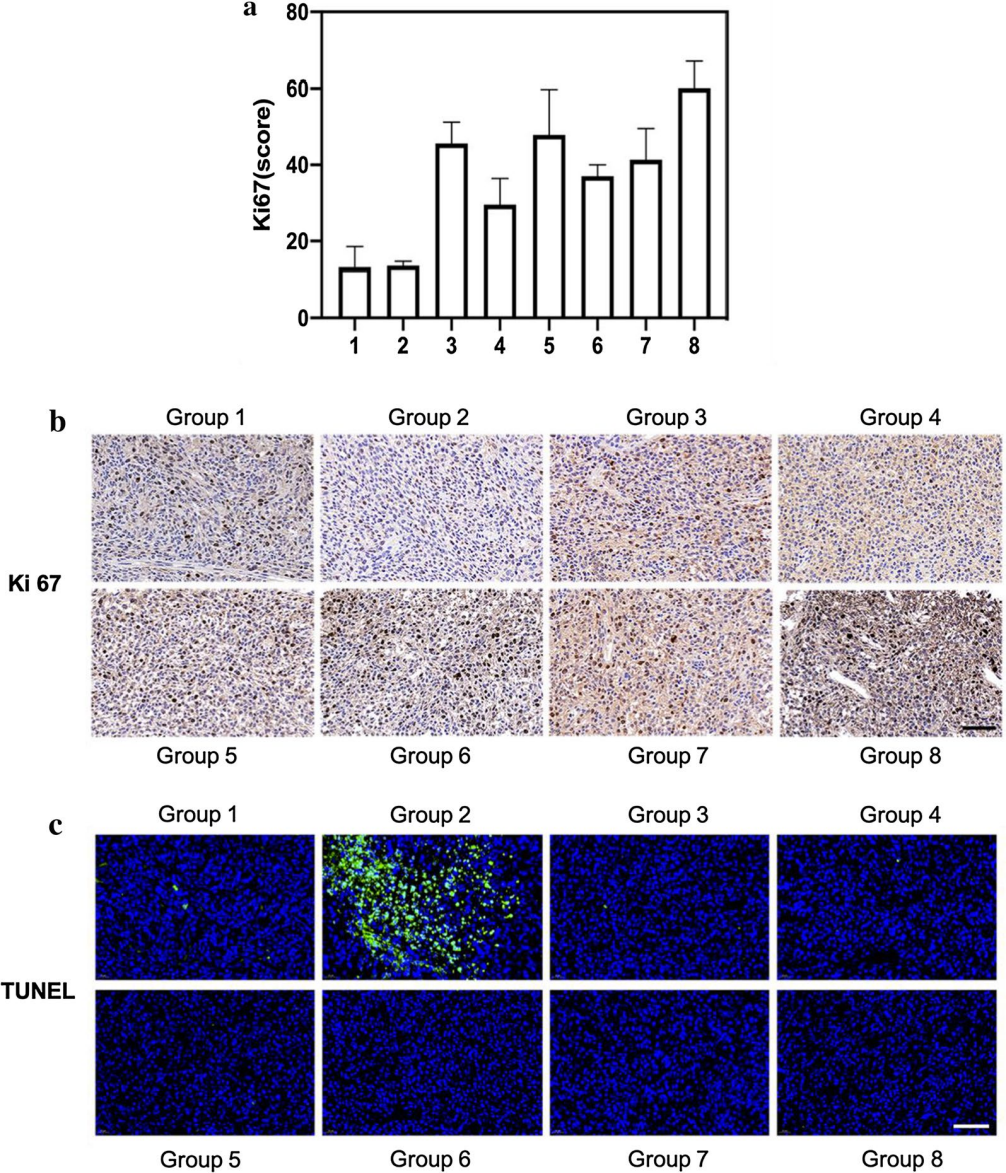
**a** The expression of Ki-67 in Group 1–8. **b**, **c** Ki-67 and TUNEL stained images of mouse tumor tissue after administration of Group 1–8. Scale bar = 60 μm. Group 1–8 correspond to the DOX-CB@lipo-iRGD-in situ-N (+) group, the DOX-CB@lipo-pDNA-iRGD-in situ-N (+) group, the DOX-CB@lipo-iRGD-iv-N (+) group, the lipo-pDNA-iRGD-in situ-N (+) group, the DOX + CB@lipo-iRGD-iv-N (+) group, the BSH-N (+), the N (+) group, and the N (−) group

Ki67 is the most reliable index to detect tumor proliferation activity [51]. Some studies [52] have shown that Ki67 is involved in the occurrence and development of glioma, which is significantly related to the pathological grade and prognosis. It is also an important index to judge the prognosis of patients with glioma. Figure 6a, b revealed that the Ki67 expression of the first to the seventh group decreased compared with the eighth groups, especially the first and the second group. The results indicated that our treatment can effectively inhibit tumor cell proliferation, especially after combined treatment. TUNEL staining analysis was carried out on the tumor tissues of mice in groups 1–8. It can be found from Fig. 6c that compared with other groups, the second group, DOX-CB@lipo-pDNA-iRGD treated mice caused significant apoptosis of tumor cells (green). It is further confirmed that our nano-drug inhibits tumor growth by inducing apoptosis.

As for the morphology of spleen tissue (Additional file 1: Fig. S20), compared with the control group 9, there was no significant change in spleen tissue in the second to and the fifth group. In the sixth group, the BSH group, the spleen tissue showed obvious degeneration, the germinal center decreased. It shows that the spleen of mice in BSH group has a toxic reaction, while the spleen toxicity of the nanoliposome is low. Meanwhile, the treatment group also caused less damage to the heart, liver, lung and kidney (Additional file 1: Fig. S21).

## Conclusion

Based on the research of tumor-targeting nano-drug delivery system, we constructed iRGD modified multifunctional nanoliposome drug delivery system (DOX-CB@lipo-pDNA-iRGD) by combining the nuclear tropism new boron-containing agent (DOX-CB) with the CD47 blocking immunotherapy, followed by the exploration of the therapeutic effect of boron neutron capture immuno-chemotherapy on glioma in situ. The multifunctional nanoliposome presented higher ratio of gene recombination efficiency and transfection efficiency on GL261 cells than the commercialized transfection vectors Lipofectamine® 2000 and PEI with significantly lower cytotoxicity. After transfection, the expression of CD47 in GL261 cells was significantly decreased, and the phagocytosis of macrophages was effectively increased. The multifunctional nanoliposome also showed good brain targeting in GL261 in situ glioma mouse model. By using one liposome material, combined treatment with boron neutron capture and immunization could be well achieved simultaneously. Therefore, we developed a highly integrated nanodrug delivery system which was capable of elevating the survival rate of tumor bearing mice with prominent curative effects, providing a promising strategy for glioma therapy.

## Experimental section

### Materials

Borocaptate Sodium (BSH) was purchased from Katchem Co., Ltd. (Prague, Czechoslovakia). Doxorubicin hydrochloride (DOX-HCl) and ixazeomib was purchased from Dalian Meilun Biotechnology Co., Ltd. (Dalian, China). iRGD (CRGDKGPDC) were customized from GL Biochem Co., Ltd. (Shanghai, China). Plasmid was customized from GenePharma Co., Ltd. (Shanghai, China), and its sequence is showed in Additional file 1: Fig. S22. 1-Bromomethyl-o-carborane (CB) was purchased from YL Biochem Co., Ltd. (Zhengzhou, China). 1,2-Dioleoyl-3-trimethylammonium-propane chloride (DOTAP), 2-dioleoyl-sn-glycero-3-phosphoethanolamine (DOPE), *N*-(carbonyl-methoxypolyethyleneglycol 5000)-1,2-distearoyl-sn-glycero-3-phosphoethanolamine (DSPE-PEG5000) was purchased from CordenPharma Co., Ltd. (Switzerland). Lipo 2000 from Life Technologies was purchased from Thermo Fisher Scientific Co., Ltd. (Shanghai, China). DPEC water was purchased from TianGen Biochem Co., Ltd. (Beijing, China). Polyethyleneimine (PEI, MW 25KDa) was purchased from Sigma-Aldrich (USA). DAPI, Lyso-Tracker Red, Firefly Luciferase Reporter Gene Assay Kit and BCA Protein Assay Kit were purchased from Beyotime Co., Ltd. (Shanghai, China). APC anti-mouse CD47 antibody (MIAP301, Santa Cruz, USA). EasyPure® HiPure PlasmidmaxiPrep Kit was purchased from TransGen Biotechnology Co., Ltd. (Beijing, China). Nuclear Extraction Kit was purchased from keygene Biochem Co., Ltd. (Nanjing, China). The NT50 D-T neutrongenerator for BNCT was kindly kindly provided by Shiwei Jing’s lab, School of Physics, Northeast Normal University. All other materials and solvents were of reagent grade and used as received.

### Synthesis of DOX-CB

DOX-HCl (300 mg, 0.5172 mmol) and triethylamine (TEA, 215 μL, 1.5517 mmol) were completely dissolved with 10 mL anhydrous DMSO and then kept stirring in vacuum for 2 h at room temperature. DOX was obtained by alkalization. Weigh 0.5172 mmol of CB and put it into the above reaction system, stir it for 48 h at room temperature. The mixture was dialyzed (the MWCO of dialysis bag was 500) for 36 h in DMSO until the dialysate became clear, then freeze-dried to obtain the solid powder of DOX-CB.

### Preparation of DOX-CB@lipo, lipo-pDNA and DOX-CB@lipo-pDNA

The plasmid was was extracted and purified by EasyPure®hiPure PlasmidmaxiPrep Kit under the guidance of the instructions. Liposomes comprising DOTAP, DOPE, CHOL and DSPE-PEG5000 in the molar ratio of 0.8:1:0.5:0.023 were prepared using the lipid film hydration method; 30 mg of all lipids were mixed with 50 mL chloroform. The lipid film was prepared in a rotary evaporator. The lipid film was then hydrated with 5 mL 5% sucrose solution (containing DEPC) for 30 min at 50 °C under shaking. The resulting liposomes were sonicated in a bath-type sonicator for 30 min and then prepared by the probe supersonic method at ice water bath (3 min, 30% power). Weigh the 3 mg DOX-CB and 30 mg of all lipids mentioned above, fully dissolved with 50 mL chloroform and repeat the procedure of liposome synthesis above. Then the DOX-CB@lipo was obtained.

The plasmid was diluted with DEPC water, and mixed with the empty cationic liposome solution (containing DEPC) in equal volume. After continuous blowing for 1 min and standing at room temperature for 20 min, the cationic liposome-plasmid complex (lipo-pDNA) was formed. DOX-CB@lipo-pDNA was obtained in this method analogously.

### Preparation of complex of nanoliposome-peptide

The iRGD was accurately weighed and prepared into solution, then mixed with the prepared nanoliposome solution, and stand at room temperature for 30 min. The complex of nanoliposome-peptide (lipo-iRGD, lipo-pDNA-iRGD, DOX-CB@lipo-iRGD, DOX-CB@lipo-pDNA-iRGD) can be obtained. The molar ratio of iRGD and nanoliposome is 5%.

### Characterization of multifunctional nanoliposomes

According to the above procedures, lipo-pDNA with different lipo/pDNA(/N/P) ratios (0, 1, 2, 3, 4, 5, 10, 20, 30, 40 and 50) were prepared. The particle size, zeta, PDI and other characteristic parameters of each sample were measured by DLS (Zetasizer90, Malvern Instruments Ltd). Each sample were measured three times in parallel.

Liposome, DOX-CB@lipo, DOX-CB@lipo-iRGD, DOX-CB@lipo-pDNA(N/P = 2), DOX-CB@lipo-pDNA-iRGD (N/P = 2) were also characterized according to the above method. The appearance characteristic was observed by TEM (H-7650, Hitachi Corporation).

### Cell lines

The cancer cell lines GL261 was purchased from Beina Chuanglian Biotechnology Institute (Beijing, China) and the luciferase transfected GL261 (Luc-GL261) were cultured in complete growth media (DMEM medium supplemented with 10% fetal bovine serum, 100 U/mL penicillin, and 100 μg/mL streptomycin) at 37 °C in a humid atmosphere maintained of 5% CO_2_.

### Cell viability assay

GL261 Cells were seeded in 96-well plates with a concentration of 3.0 × 10^3^ cells/well, respectively. After 24 h hatch in cell culture incubator, different polymeric formulations (lipo-pDNA, lipo-pDNA-iRGD, DOX-CB@lipo-pDNA, DOX-CB@lipo-pDNA-iRGD with different N/P ratio 0.4, 1, 1.6, 2, 4, 10, 20, 40) were added into each well in various concentrations for 48 h incubation followed by Cell Counting Kit-8 (CCK8, Beyotime, Shanghai, China). And cell viability was calculated as percentages by compared with control (lipo2000 with plasmid with different N/P ratio 2, 3, 4, 5 referring to the Life Technologies). The optical density at 450 nm (OD_450_ nm) was measured using a multiwell plate reader (ELX800, Biotek, USA). Each well was repeated at least 6 times, and cell proliferation was presented as mean ± SD.

### Gene recombination assay

A series of lipo-pDNA (N/P ratio 1, 1.6, 2, 4) and lipo2000-pDNA (2) were prepared according to the above experimental procedures. Plasmid solution (100 μg/mL) and PEI solution (130 μg/mL) were mixed by equal volume to obtain the PEI-pDNA complex.

GL261 Cells were seeded in 24-well plates with a concentration of 5.0 × 10^4^ cells/well, respectively. After 18 h hatch in cell culture incubator, PEI-pDNA, lipo2000-pDNA (2), lipo-pDNA (N/P = 2) were added into each well for 6 h incubation. Then incubate for 48 h in fresh growth media and the expression of GFP was observed under inverted fluorescence microscope.

### Western blot analysis

To examine the gene transfection efficiency of different preparations, GL261 Cells were seeded in 6-well plates with a concentration of 1.0 × 10^5^ cells/well and incubated for 18 h. 160 μL naked pDNA, lipo2000-pDNA (2), lipo-pDNA (N/P = 2) and lipo-pDNA (N/P = 4) of an equivalent pDNA dose of 2.5 μg/μL with 2 mL serum-free medium were added into each well for 6 h incubation. Fresh serum containing complete culture medium was used to continue the culture for 72 h. Then we collected GL261 cells and obtained cellular proteins using T-PER Tissue Protein Extraction Reagent (Thermo Pierce). The total protein concentration was then quantified by a BCA protein assay kit. Western blot tests were processed in the standard fashion, and analyzed quantitatively protein expressions by the ImageJ software. The following antibodies were used in the procedure: CD47 (ab175388, Abcam), GAPDH (ab181602, Abcam), and goat anti-rabbit IgG (H + L) secondary antibody (31210, Thermo Pierce).

### Determination of gene transfection efficiency

GL261 Cells were seeded in 6-well plates with a concentration of 5.0 × 10^4^ cells/well and incubated for 18 h. Then washed by PBS three times and then coincubated for another 6 h, respectively, with 40 μL lipo2000-pDNA (2), lipo-pDNA (N/P = 2), lipo-pDNA (N/P = 4) of an equivalent pDNA dose of 2.5 μg/μL and 500 μL serum-free medium. Fresh serum containing complete culture medium was used to continue the culture for 24 h. In the experiment, pure culture medium blank control group and plasmid incubation control group were set.

After transfection, the cells were washed by PBS for 3 times, then 200 μL cell lysis solution was added in each well and shaked for 40 min at room temperature. After carefully blowing the lysate repeatedly, the liquid was centrifuged at 4 °C for 20 min, and the rotating speed was 14,000 rpm. The supernatant was moved into the new EP tube. Add the equal volume of Firefly Luciferase Reporter Gene Assay Kit into EP tube, mix evenly and determined the relative light unit (RLU) by the chemiluminescence instrument (E6080, Promega, USA). In the same time, BCA Protein Assay Kit was used to determine the corresponding protein concentration. The relative light intensity per mg protein (RLU per mg protein) was used to evaluate the transfection efficiency of each sample.

### Flow cytometry assay

To detect the expression of CD47 after transfection, GL261 Cells were seeded in 6-well plates with a concentration of 1.0 × 10^5^ cells/well and incubated for 18 h. 160 μL naked pDNA, lipo2000-pDNA (2), lipo-pDNA (N/P = 2) and lipo-pDNA (N/P = 4) of an equivalent pDNA dose of 2.5 μg/μL with 2 mL serum-free medium were added into each well for 6 h incubation. Compared with control (without polymers incubation and with only plasmid incubation). Then incubate for 72 h in fresh growth media. The experiments were repeated 3 times.

After digestion and centrifugation, the cells were incubated with anti-mouse CD16/32 on ice for 10 min, washed with PBS for 3 times, and incubated with APC anti-mouse CD47 antibody on ice for 30 min, then washed with PBS for 3 times. The expression of CD47 in each sample was detected by flow cytometry.

### Cellular uptake and distribution

To determine the cellular uptake of DOX, GL261 cells were seeded into glass bottom dishes (20 mm, Nest) at a cell density of 1.0 × 10^4^ cells/dish, incubated for 24 h, washed by PBS and then coincubated for another 6 h, respectively, with free DOX and DOX-CB of an equivalent DOX dose of 3.5 μM. After washing with cold PBS for three times, the cells were fixed in 4% paraformaldehyde, incubated with DAPI for 10 min, and rinsed with PBS for three times. The DAPI and intracellular DOX were intuitively observed by laser scanning confocal microscope (LSCM, Zeiss LSM 800, Germany). The fluorescence channels for DOX were kept at the same laser intensity so as to quantify cellular uptake of DOX by an US National Institutes of Health-ImageJ software. For cellular distribution of different preparations, the cells were seeded into dishes, and incubated with free DOX (1 μg/mL), DOX + CB, DOX-CB, DOX-CB@lipo, DOX-CB@lipo-pDNA-iRGD of the equivalent DOX dose, treating with or without ixazeomib for 2, 4, and 8 h. Samples were then fixed, stained with DAPI, and visualized by LSCM eventually.

### Determination of boron content ratio in nucleus and cytoplasm

The tumor cells GL261 in logarithmic growth phase were evenly seeded in a 150 mm diameter cell culture dishes, incubated for 16 h, washed by PBS and then coincubated for another 3 h, respectively, with free DOX, free CB, DOX + CB, DOX-CB@lipo, DOX-CB@lipo-pDNA-iRGD of the equivalent boron dose of 3.5 μg/mL. After washing with cold PBS for three times, the cells were counted and collected, at the same time part of the samples were handled with the Nuclear Extraction Kit at low temperature, and treated with nuclear staining agent. Finally, add an appropriate amount of concentrated nitric acid and hydrogen peroxide mixed solution (V: V = 3:1) to the obtained cells and nuclei, heat them with wet method at 70 °C, and digest them for more than 24 h. The boron concentration was determined by ICP-MS and the ratio of boron content in nucleus and cytoplasm was calculated.

### Phagocytic assay

GL261-GFP was transfected with lipo2000-pDNA (2) and lipo-pDNA (N/P = 2) as the previous procedure and collected. In the experiment, pure culture medium blank control group and plasmid incubation control group were set. The primary mouse BMDMs were extracted from C57BL/6 mice using the previous method [53, 54] and then resuspended in complete culture medium and evenly inoculated in confocal dish. After 5–7 days of stimulation and differentiation, they were replaced with fresh culture medium. The cells were fixed by 4% paraformaldehyde for 20 min, handled with 0.25% Triton X-10 for 20 min, then blocked with goat serum for 25 min. The samples were stained with primary antibodies CD47 (ab175388, Abcam) conjugated to F4/80 in 1:30 dilution. Secondary antibody goat anti-mouse IgG (ab31160, Abcam) was utilized in 1:200 dilution and stained with DAPI before observation. The fluorescence images were taken by inverted confocal microscope to observe the phagocytosis of macrophages to tumor cells.

### Mice and animal models

C57BL/6 mice (male, 5-week-old) purchased from Slaccas (Shanghai, China) were adaptive fed for more than 1 week for subsequent experiments. The animals were maintained under standard housing conditions and all related experiments were conducted followed by the guidelines which has been evaluated and approved by the Ethics Committee of Zhejiang University.

Orthotopic Glioma Mice Model: To establish the glioma model, 5 week-old male C57BL/6 mice (about 20 g) were anesthetized by intraperitoneal injection of 0.08 mL/10 g 1% pentobarbital solution. After the mice were fixed and the surgical area was exposed, 3 × 10^5^ cells/5 μL Luc-GL261 cells were trypsinized and resuspended in sterile PBS were slowly injected into the left corpus striatum (2 mm lateral, 1 mm posterior to the bregma, and 3.0 mm in depth) by a stereotactic fixation device using a mouse adaptor. Six days later, the orthotopic glioma mice model were imaged and evaluated by IVIS spectrum (PerkinElmer, USA) after 5 min postintraperitoneal injection of d-luciferin (150 mg∙kg^−1^, Gold Biotechnology, USA). The MRI (4.7 T/30 cm Bruker Biospec scanner, Germany) was observed too. The T/N ratios and BNCT experiments were executed in the orthopotic glioma mice model. The T/N ratios were the ratios of the boron concentration in the tumor to that in the surrounding normal tissue measured by ICP-MS.

### In vivo anti tumor effect of BNCT combined immunochemotherapy

The orthotopic glioma mice model were randomly divided into nine groups: (1) DOX-CB@lipo-iRGD-in situ-N (+): On day 6 to day 8, the mice were treated with DOX-CB@lipo-iRGD by intratumoral injection, after the injection for about 24 h, all the mice were irradiated with the thermal neutron beam for 2 h at the neutron irradiator (The neutron generator ion source voltage is 1879 V, the ion source current is 0.208 mA, the accelerator voltage is 90 kV, and the thermal neutron yield is about 1.58% × 10^8^/s). (2) DOX-CB@lipo-pDNA-iRGD-in situ-N (+): On day 6 to day 8, the mice were treated with DOX-CB@lipo-pDNA-iRGD by intratumoral injection, after the injection for about 24 h, all the mice were irradiated with the thermal neutron beam for 2 h at the neutron irradiator. On the day 13, day 16, day 19, the mice were in situ injected with 10 μL lipo-pDNA-iRGD. (3) DOX-CB@lipo-iRGD-iv-N (+): On day 6 to day 8, the mice were treated with DOX-CB@lipo-iRGD by intravenous injection, after the injection for about 24 h, all the mice were irradiated with the thermal neutron beam for 2 h at the neutron irradiator. (4) lipo-pDNA-iRGD-in situ-N (+): On day 6 to day 8, the mice were treated with lipo-pDNA-iRGD by intratumoral injection, after the injection for about 24 h, all the mice were irradiated with the thermal neutron beam for 2 h at the neutron irradiator. On the day 13, day 16, day 19, the mice was in situ injected with 10 μL lipo-pDNA-iRGD. (5) DOX + CB@lipo-iRGD-iv-N (+): On day 6 to day 8, the mice were treated with DOX + CB@lipo-iRGD by intravenous injection, after the injection for about 24 h, all the mice were irradiated with the thermal neutron beam for 2 h at the neutron irradiator. (6) BSH-N (+): On day 9, the mice were treated with 100 μL BSH by intravenous injection, after the injection for about 2 h, all the mice were irradiated with the thermal neutron beam for 2 h at the neutron irradiator. (7) N (+): On day 9, all the mice were irradiated with the thermal neutron beam for 2 h at the neutron irradiator. (8) N (−): Blank control group was made after modeling, without irradiation. (9) sham: 5 μL PBS intracranial injection was used as the sham operation control group, without irradiation. The concentrations of DOX-CB, DOX + CB was 0.3 mg/mL, the concentrations of BSH was 20 mg/mL, the concentrations of plasmid was 100 μg/mL, and the ratio of N/P was 2. The survival curve and average weight change curve of each experimental group were recorded and drawn.

### Histopathological evaluation

For histological analysis, the brain, heart, liver, spleen, lung, and kidney samples of the mice model in the different groups were fixed in 4% paraformaldehyde for 24 h and washed twice with PBS. Paraffin-embedded tissue sections were stained with H&E and observed on a microscope. The brain tumors were also detected by immunohistochemistry.

### Statistics

Statistical analysis was performed using GraphPad Prism 8 (GraphPad Software, CA, USA). Data were presented as means ± S.D. Statistical evaluation of differences between experimental groups was performed by one-way ANOVA followed by Tukey’s multiple comparisons test. Statistical significance was considered at least at p < 0.05.

## Supplementary Information

**Additional file 1.** Concludes the results of the characterization of multifunctional nanoliposomes and DOX-CB and the results of cellular distribution and the cell viability of DOX-CB. The results of additional animal experiments such as evaluation of the GL261-orthotopicglioma in the C57BL/6 mouse model and HE staining of different organs.

## Data Availability

All data generated or analysed during this study are included in this published article.
