# Supplementary material for: Therapeutic nucleus-access BNCT drug combined CD47-targeting gene editing in glioblastoma
Source: J Nanobiotechnology. 2022 Mar 4;20:102. doi: 10.1186/s12951-022-01304-0 (PMC8895533; doi:10.1186/s12951-022-01304-0)
Supplement: Supplementary file 1 — Additional file 1. Concludes the results of the characterization of multifunctional nanoliposomes and DOX-CB and the results of cellular distribution and the cell viability of DOX-CB. The results of additional animal experiments such as evaluation of the GL261-orthotopicglioma in the C57BL/6 mouse model and HE staining of different organs. [file 12951_2022_1304_MOESM1_ESM.docx]

**Supporting Information**

**Therapeutic nucleus-access BNCT drug combined CD47-targeting gene editing in glioblastoma**

*Jiejian Chen^A,#^, Qi Dai^B,#^, QiYao Yang^C^, Xiaoyan Bao^D^,Yi Zhou^E^, Haiqing Zhong^F^, Linjie Wu^G^, Tiantian Wang^H^, Zhicheng Zhang^I^, Yiying Lu^J^, Zhentao Zhang^K^, Mengting Lin^L^, Min Han^M,*^,Qichun Wei^N,*^*

A*. Jiejian Chen*

Institute of Pharmaceutics, Zhejiang Province Key Laboratory of Anti-Cancer Drug Research, College of Pharmaceutical Sciences, Zhejiang University, Hangzhou 310058, P.R. China

Department of Radiation Oncology, Key Laboratory of Cancer Prevention and Intervention, The Second Affiliated Hospital, College of Medicine, Zhejiang University, Hangzhou 310058, China.

Department of Medical Oncology, Guangzhou First People's Hospital, School of Medicine, South China University of Technology, Guangzhou, 510180, Guangdong, China.

B. *Qi Dai* C. *QiYao Yang* I. *Zhicheng Zhang* N*. Qichun Wei*

Department of Radiation Oncology, Key Laboratory of Cancer Prevention and Intervention, The Second Affiliated Hospital, College of Medicine, Zhejiang University, Hangzhou 310058, China.

D. *Xiaoyan Bao* E*. Yi Zhou* F*. Haiqing Zhong* G*. Linjie Wu* H*. Tiantian Wang* J*. Yiying Lu* K*. Zhentao Zhang* L*. Mengting Lin* M*, Min Han*

Institute of Pharmaceutics, Zhejiang Province Key Laboratory of Anti-Cancer Drug Research, College of Pharmaceutical Sciences, Zhejiang University, Hangzhou 310058, P.R. China

* Corresponding author: qichun_wei@zju.edu.cn (Qichun Wei), hanmin@zju.edu.cn (Min Han).

#: these two authors contributed equially to this work.

**Materials and Methods**

Fetal bovine serum (Gibco, USA), penicillin streptomycin double antibody mixture (Gibco, USA), RPMI-1640 cell culture medium (Wuhan Boster Biotechnology Co., Ltd., China), DMEM high sugar liquid culture medium (Gibco, USA), trypsin (containing 0.02% EDTA, Wuhan Boster Biotechnology Co., Ltd., China), potassium bromide (Sigma, USA), 4% paraformaldehyde solution (Beijing solarbio Technology Co., Ltd., China), Concentrated nitric acid and 30% hydrogen peroxide solution (Sinopharm Chemical Reagent Co., Ltd., China).

U87 and C6 cell lines were purchased from the cell resource center of iCell Bioscience Inc. (Shanghai, China)

**Characterization of multifunctional nanoliposomes**

According to the above procedures, lipo-pDNA with different lipo/pDNA(N/P) ratios (0, 1, 2, 3, 4, 5, 10, 20, 30, 40 and 50) were prepared.

**Agarose Gel Retardation Assay**

The binding ability of liposomes to plasmid DNA can be analysis by agarose gel electrophoresis. A series of cationic liposome-plasmid complexes (lipo-pDNA) with different N/P ratios (0.2, 0.4, 1, 1.6 and 2) were prepared, DNA marker group (M) and pure plasmid group (N) were set as controls. All samples were electrophoretic in prepared agarose gel, and then observed in gel imaging system. Then take photos to preserve experimental pictures.

**Characterization of DOX-CB.**

Using the chemical shifts of the corresponding characteristic peaks in the ^1^H-NMR spectra to analyze the structures of the DOX-CB, and the two-dimensional NOESY spectra were scanned. Using ^11^B-NMR and IR detect the structure and composition of DOX-CB. Using UV absorption spectrometer to detect the maximum UV absorption wavelength of DOX-CB and evaluate the specificity of DOX or DOX-CB. The boron concentration of DOX-CB was determined by ICP-MS.

**Cell Viability Assay**

Take tumor cells (GL261, U87, C6) in logarithmic growth stage respectively and inoculate them evenly on sterile 96 well cell culture plate with the number of 3×10^3^ cells per well then placed in the cell incubator and cultured for 24 h. Remove the old culture medium and add 100 μL fresh culture medium containing medicine (BSH group, free DOX group, DOX-CB group or free CB group). The culture medium containing medicine in each group is configured into gradient proportional concentration, and the minimum concentration of BSH group (or free CB group) is 6.25 μM. The lowest concentration in the group containing free DOX was 0.3125 μg/ mL, while the lowest concentration of DOX-CB group was 1.25 μg/mL. Place the added cell culture plate in the cell culture box for incubation for 48 h (or 24 h), suck out the drug containing culture medium, add the culture medium containing 10% CCK8, and shake the bed for 5 min after incubation for 2 h. Finally, the absorbance value (OD value) was measured on the enzyme labeling instrument, the wavelength was 450 nm, and 6 multiple holes were set for each concentration of drug.

**Intracellular fluorescence distribution experiment**

Take tumor cells (U87, C6) in logarithmic growth stage, evenly inoculate them with the density of 1×10^4^ cells per dish in a glass bottom dish for sterile confocal microscope, place them in a cell incubator and culture for 24 hours. Prepare preparations containing free DOX or DOX-CB respectively (DOX concentration is 3.5 μM) Cell culture medium. Replace the with the freshly prepared medicated culture medium and continue to incubate for 6 h. Discard the culture medium, wash it three times with PBS, fix it at room temperature with 4% paraformaldehyde for 20 min, wash it properly, add ready to use DAPI staining solution to completely cover the cells in the dish, incubate it for 10 min at room temperature, wash it three times with PBS. The cell fluorescence pictures were taken by inverted confocal microscope. The excitation wavelength of DAPI was 406 nm and that of DOX (or DOX-CB) was 488 nm.

**Magnetic Resonance Imaging of tumor bearing mice**

After 2 weeks of modeling (modeling method as mentioned in the **Mice and animal models** section in the text), the mice were anesthetized with isoflurane inhalation, placed in a 9.4 T small animal nuclear magnetic imaging instrument for brain magnetic resonance imaging (MRI), and scanned the cross-section and coronal plane of the mouse head. The main scanning parameters were TSE sequence T2WI, TR (2500.0 ms), TE (30.0 ms). Finally, the data were copied and the pictures were exported after being processed by RadiAnt DICOM Viewer software.

**Preparation and evaluation of brain tissue specimens**

Two weeks after modeling (modeling method as mentioned in the **Mice and animal models** section in the text), the mice were anesthetized with 1% Pentobarbital Sodium Solution (0.08 mL/10g body weight) and fixed on the operating table. After drawing normal saline with a 20 mL syringe, the needle tube is sleeved with a scalp needle, the needle is inserted into the left ventricle of the mouse for about 2 mm, and a small cut is cut in the right atrium with an ophthalmic scissors. The mouse heart is perfused slowly to ensure the staining effect of brain tissue sections. After the perfusion, the brain tissue of mice was carefully extracted, quickly soaked in neutral formalin, taken out for 24 hours, photographed, cut the general sample of brain tissue along the coronal section of the tumor inoculation point, photographed and stored the experimental results. Mice of the same week old were used as the normal control group.

In order to evaluate the integrity of blood-brain barrier after tumor formation in mice, the tumor bearing mouse model was established by the same operation as above. 0.1% Evans blue aqueous solution (0.1 mL) was injected into caudal vein. After 2 h, the mice were anesthetized and perfused. The mouse brain tissue was carefully removed, quickly soaked in neutral formalin, and taken out for photography for 24 h. At the same time, the brain tissue was cut along the coronal section of the tumor inoculation point, followed by HE staining, and the tumor infiltration of tissue samples was analyzed under an inverted microscope. Mice of the same week old were used as the normal control group.

In order to further evaluate the infiltration of GL261 tumor cells into brain tissue, the tumor bearing mouse model was established with GL261-GFP as the model cell and the same as the above operation. After 2 weeks of anesthesia and cardiac perfusion, the mouse brain tissue was carefully removed, fixed with neutral formalin, DAPI staining and labeling the nucleus, making sections, and taking fluorescence pictures on the machine under the fluorescence microscope.

In order to dynamically evaluate the growth of brain tissue, the tumor bearing mouse model was established with the above operations. The mice were taken out at different time points (4, 8, 15 and 20 days). HE sections were prepared after anesthesia, cardiac perfusion and neutral formalin fixation.

**Bioluminescence imaging of tumor bearing mice *in vivo***

Luc-Gl261 tumor cells were injected into mice in situ to construct an in situ glioma mouse model. Isoflurane inhalation anesthetized mice. Then the mice were transferred to the living bioluminescence imaging system, photographed and stored data. Mice of the same week old were used as the normal control group.

***In vivo* fluorescence imaging of tumor bearing mice**

GL261 *in situ* tumor bearing mice model was established. Two weeks later, the tumor bearing mice were divided into three groups and injected with 0.2 mL free DiR solution, DiR@lipo and DiR@lipo-iRGD. After 24 hours, the mice were anesthetized with isoflurane inhalation and placed in the *in vivo* imaging system for observation. The exposure times between various were consistent.

The main organs were isolated and immediately placed in the small animal *in vivo* imaging system for observation. The isolated brain tissue of mice treated with the preparation was cut along the coronal plane along the cell inoculation hole, and fluorescent pictures were taken at the same exposure time.


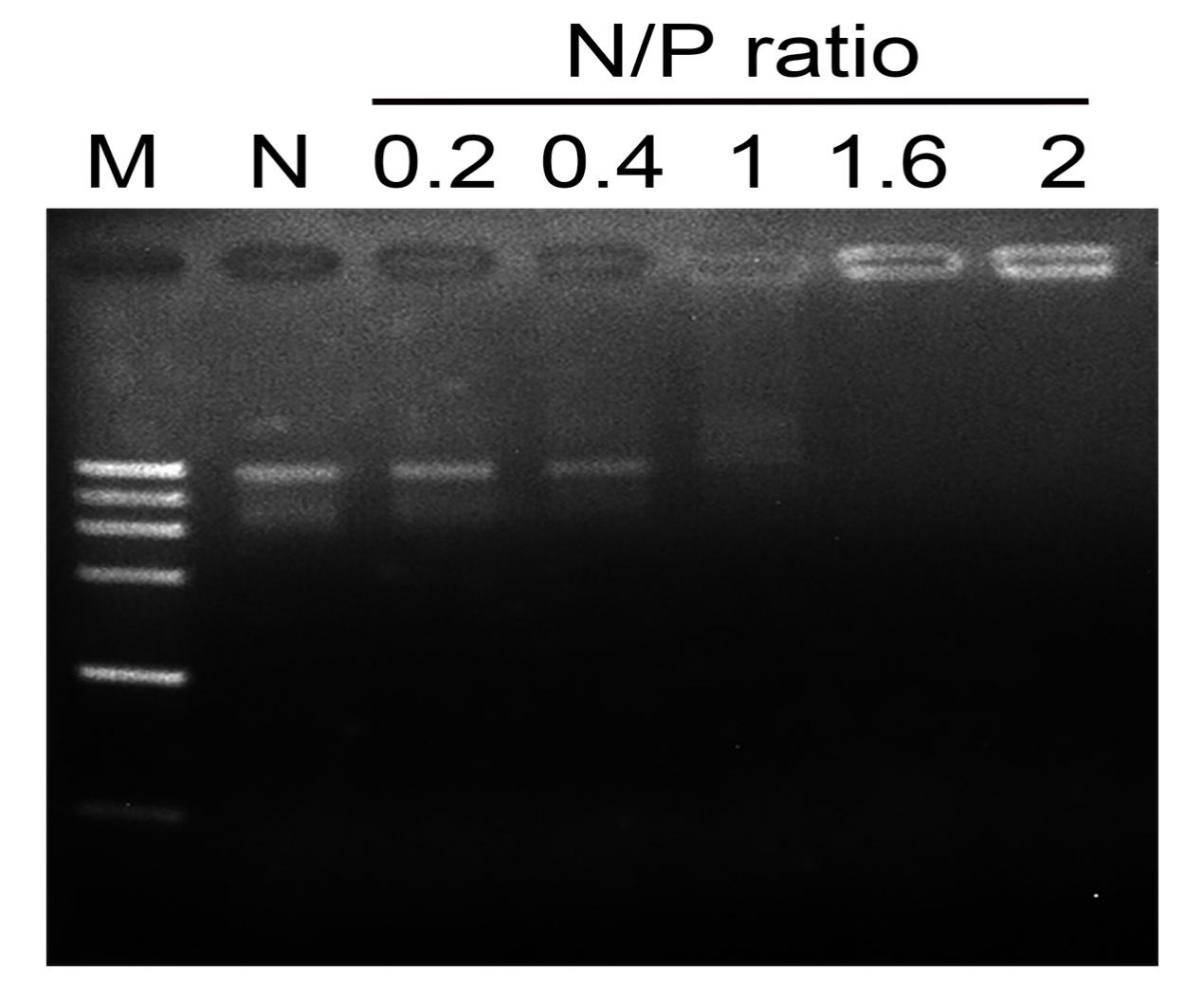


**Figure S1.** Agarose gel retardation assay performed for plasmid and liposome complexes at different N/P ratios.


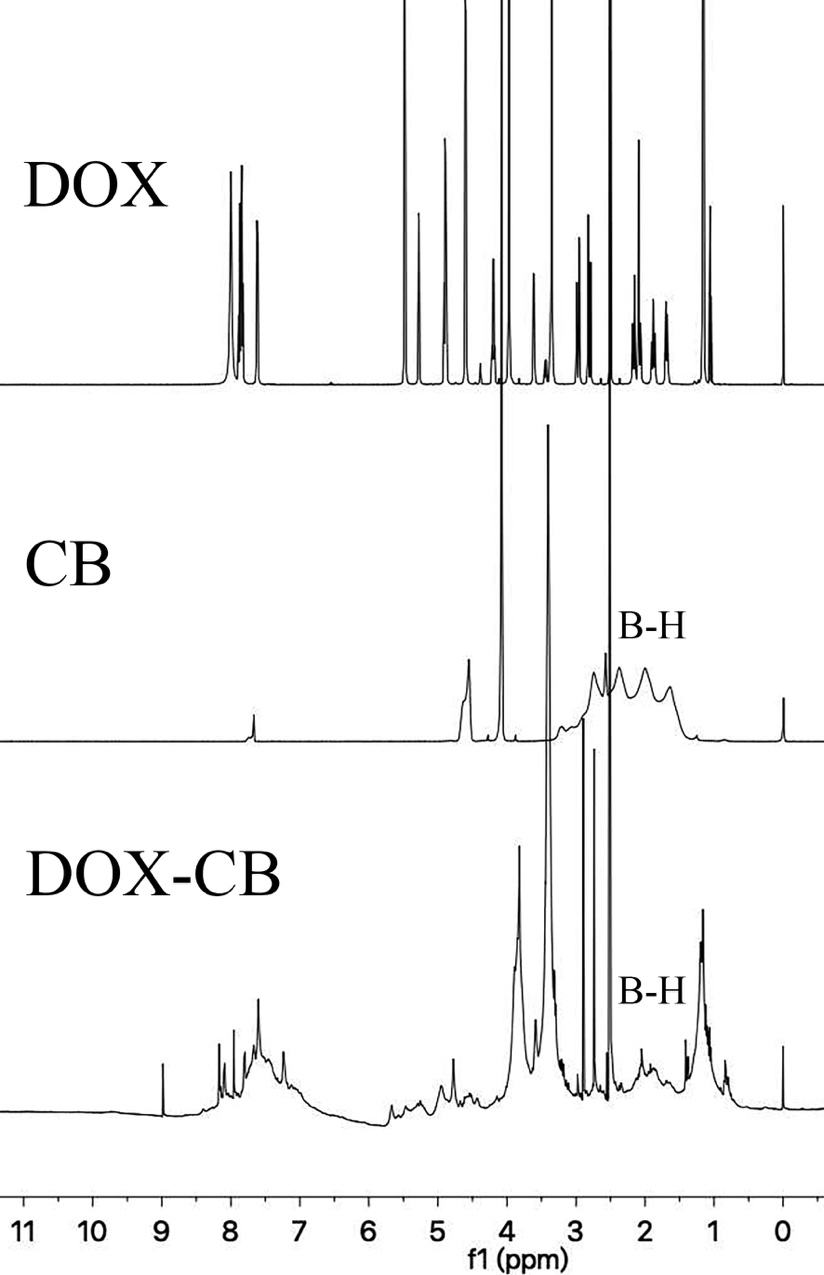


**Figure S2.** ^1^H NMR spectra of DOX, CB and DOX-CB.


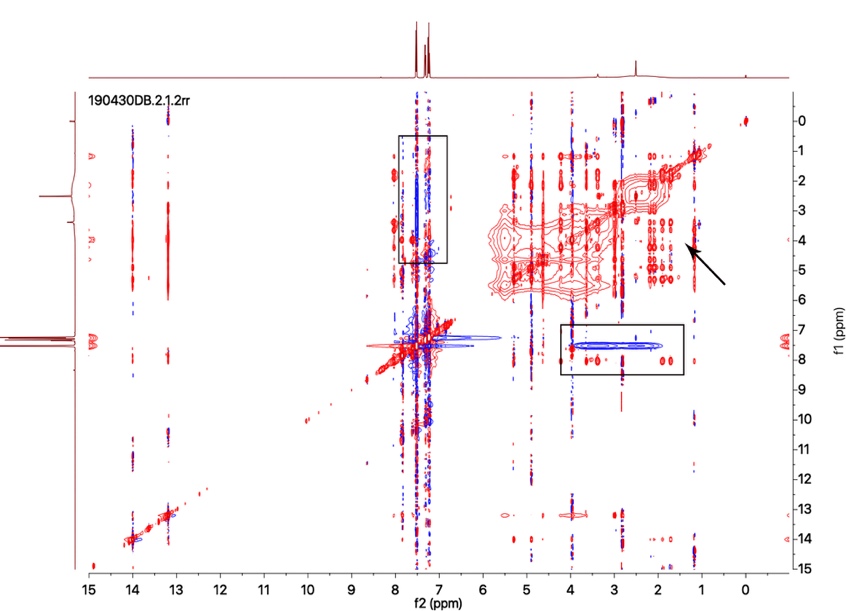


**Figure S3.** Investigation of intermolecular interactions by 2D NMR analysis.


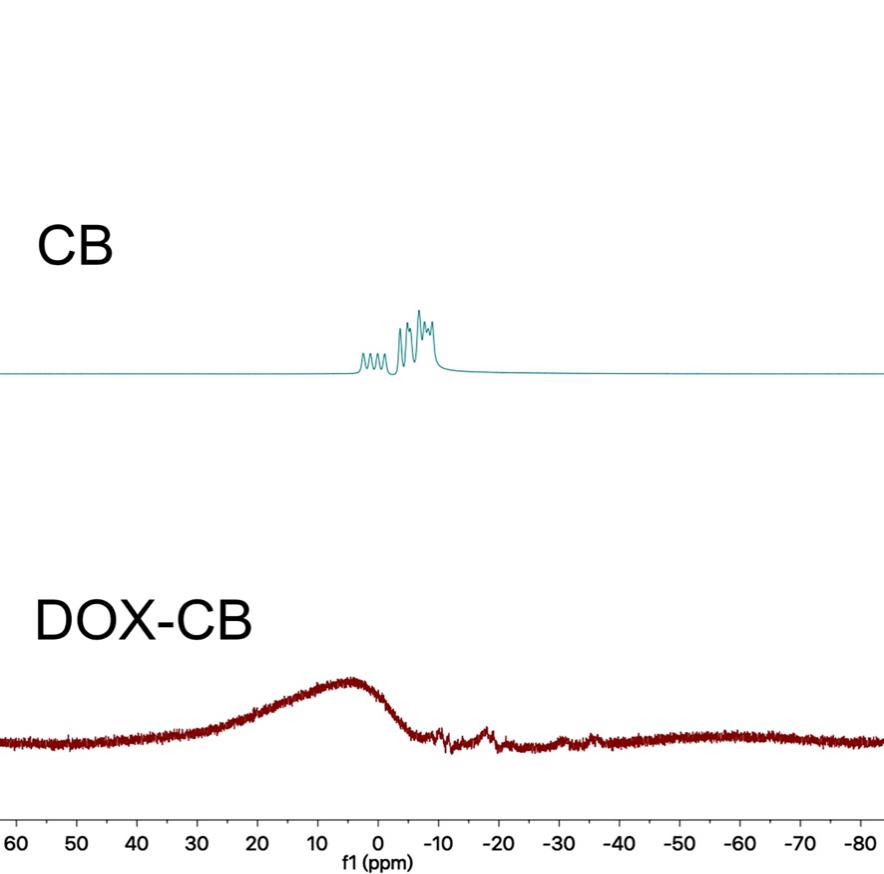


**Figure S4.** ^11^B NMR spectra of DOX, CB and DOX-CB.


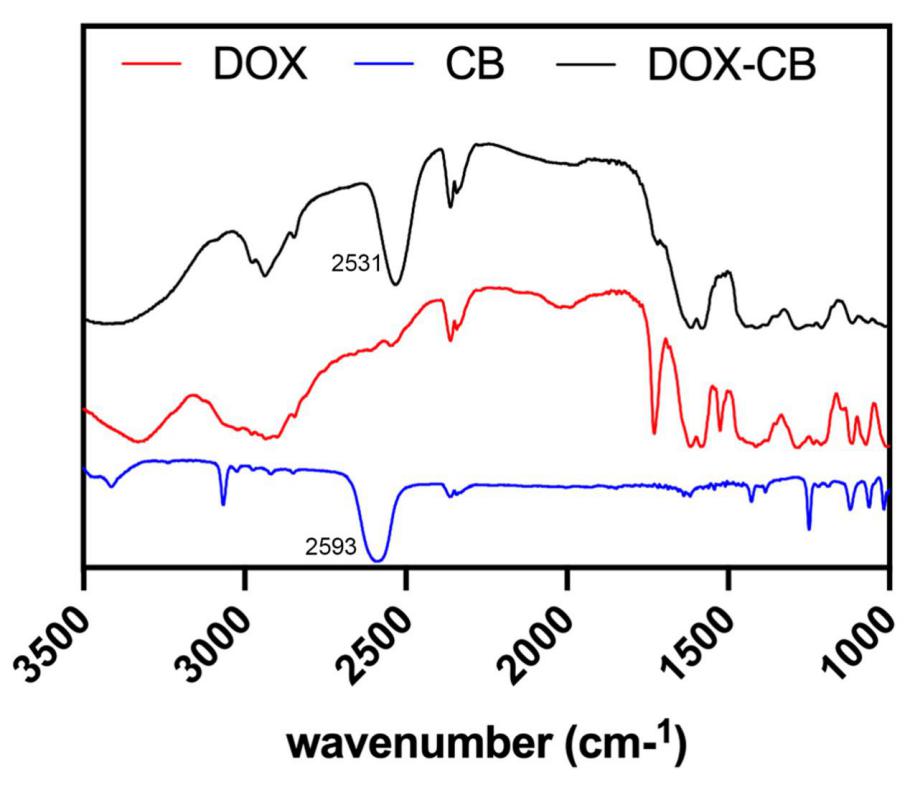


**Figure S5.** IR spectra of DOX, CB and DOX-CB.


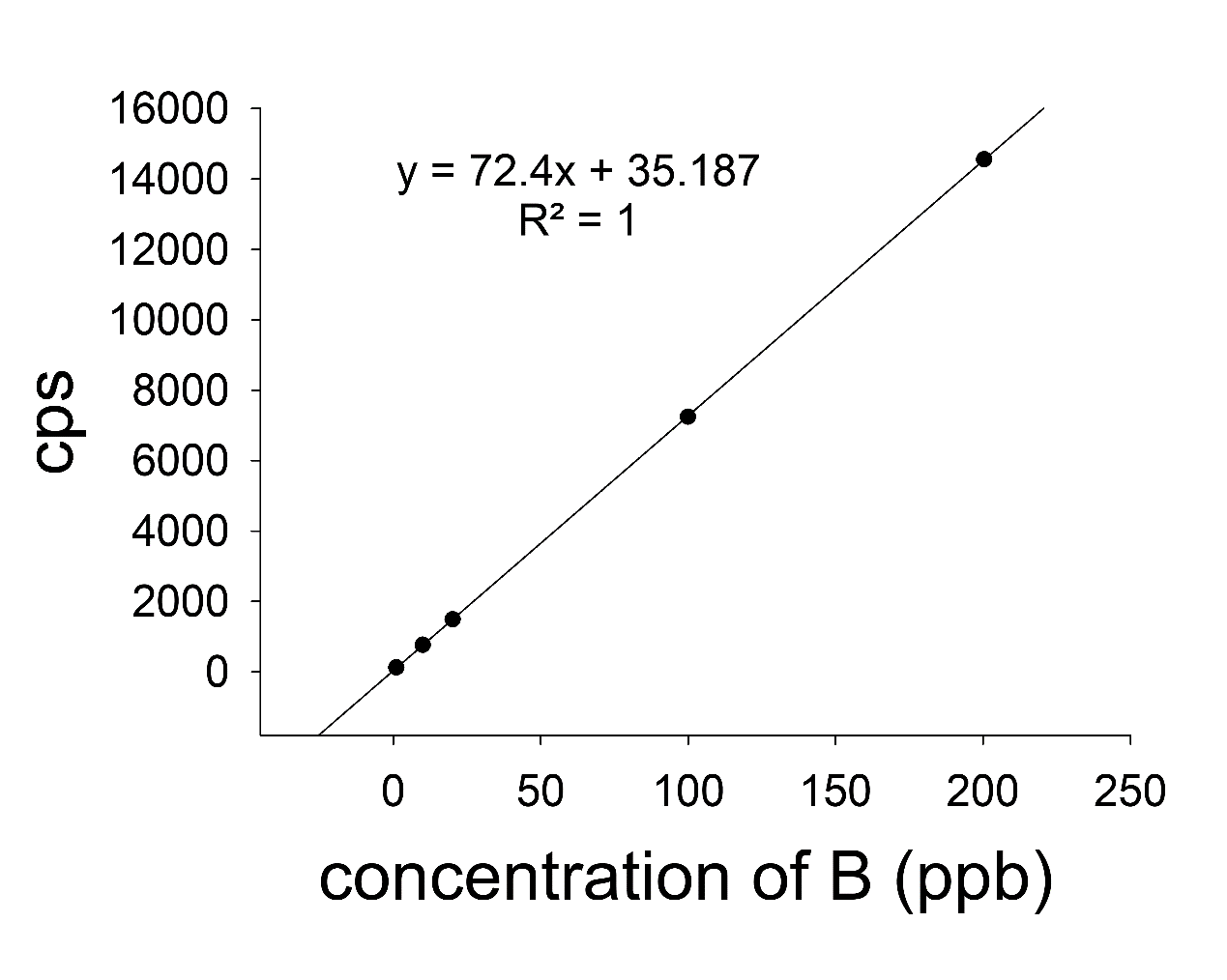


**Figure S6.** Standard curve of boron concentration measured by ICP-MS.


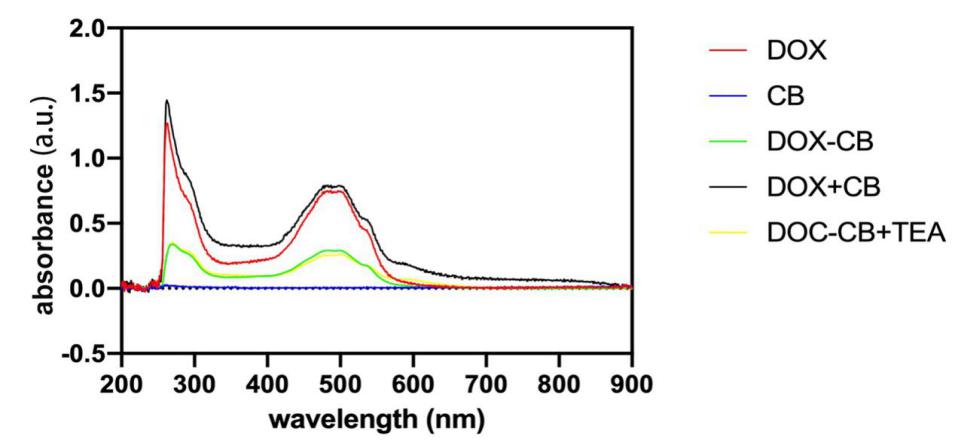


**Figure S7.** The absorption spectra of group DOX, CB, DOX-CB, DOX+CB and DOX-CB+TEA.


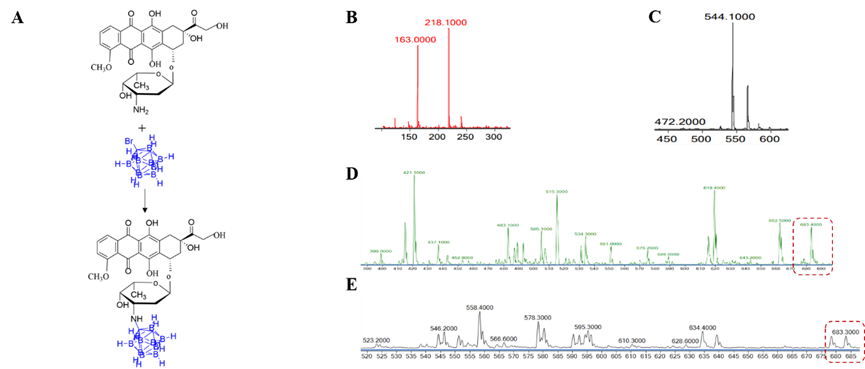


**Figure S8.** A, Synthetic scheme for the preparation of DOX-CB. B-E, The LC-MASS of CB(B), DOX(C), DOX-CB(D), DOX-CB@lipo-iRGD(E).


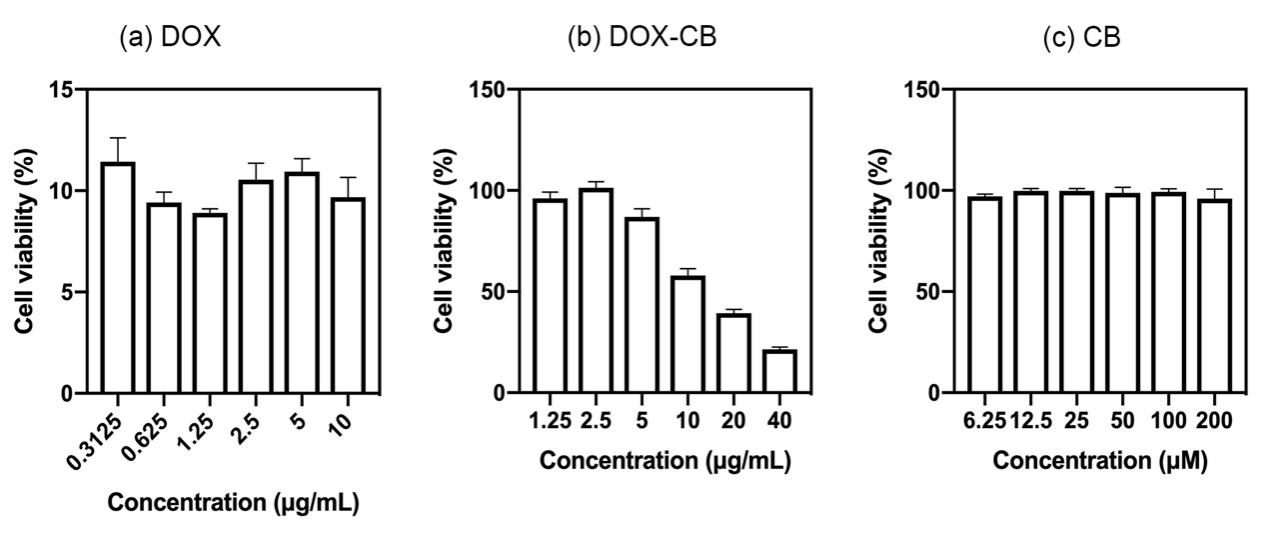


**Figure S9.** Evaluation of cell viability (CCK-8 assay) of GL261 cells after 48h of treatment with different preparations respectively. Values are expressed as mean ± SD (n  =  6).


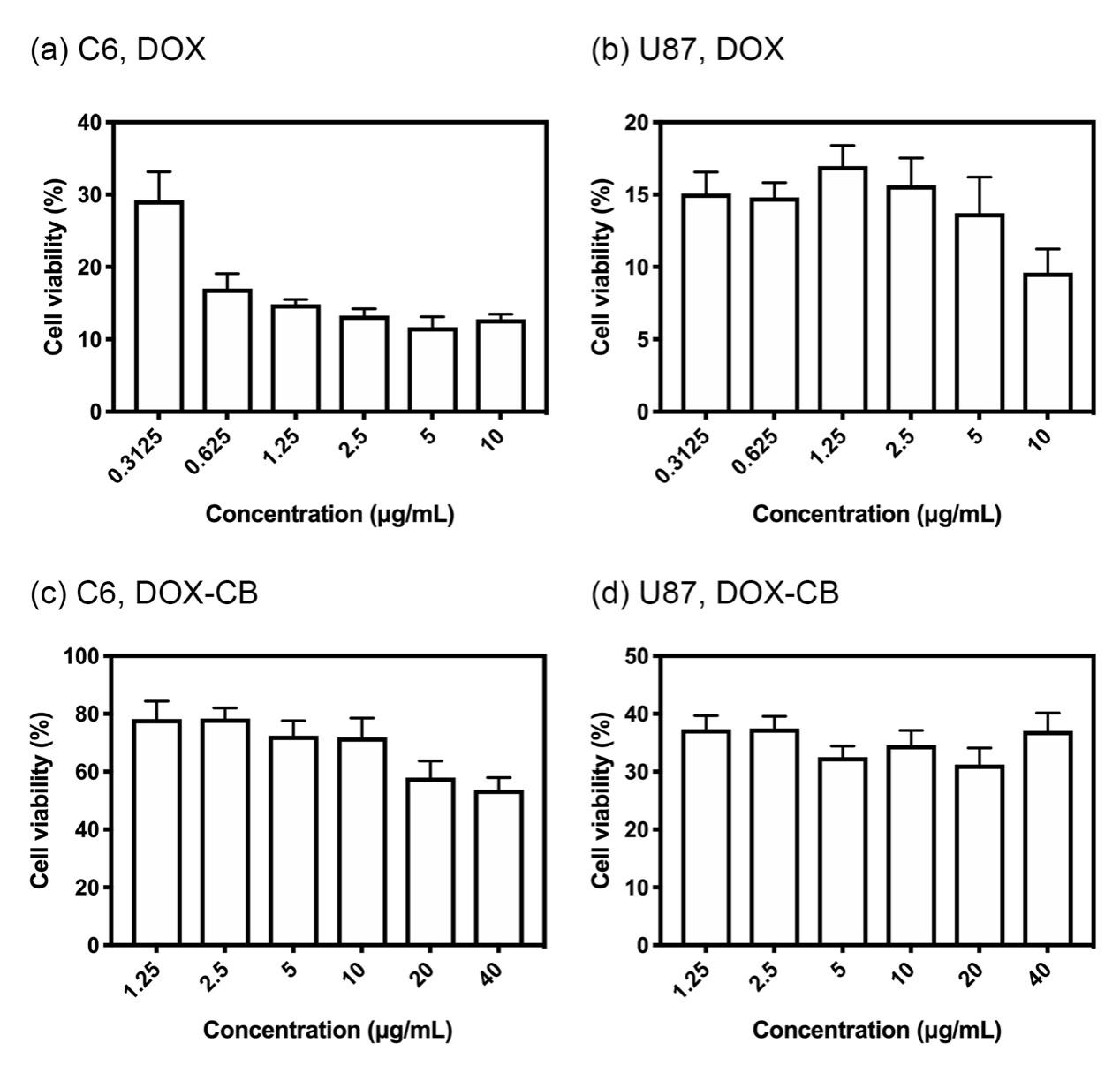


**Figure S10.** Evaluation of cell viability (CCK-8 assay) of C6 or U87 cell lines after 48h of treatment with different preparations respectively. Values are expressed as mean ± SD (n  =  6).


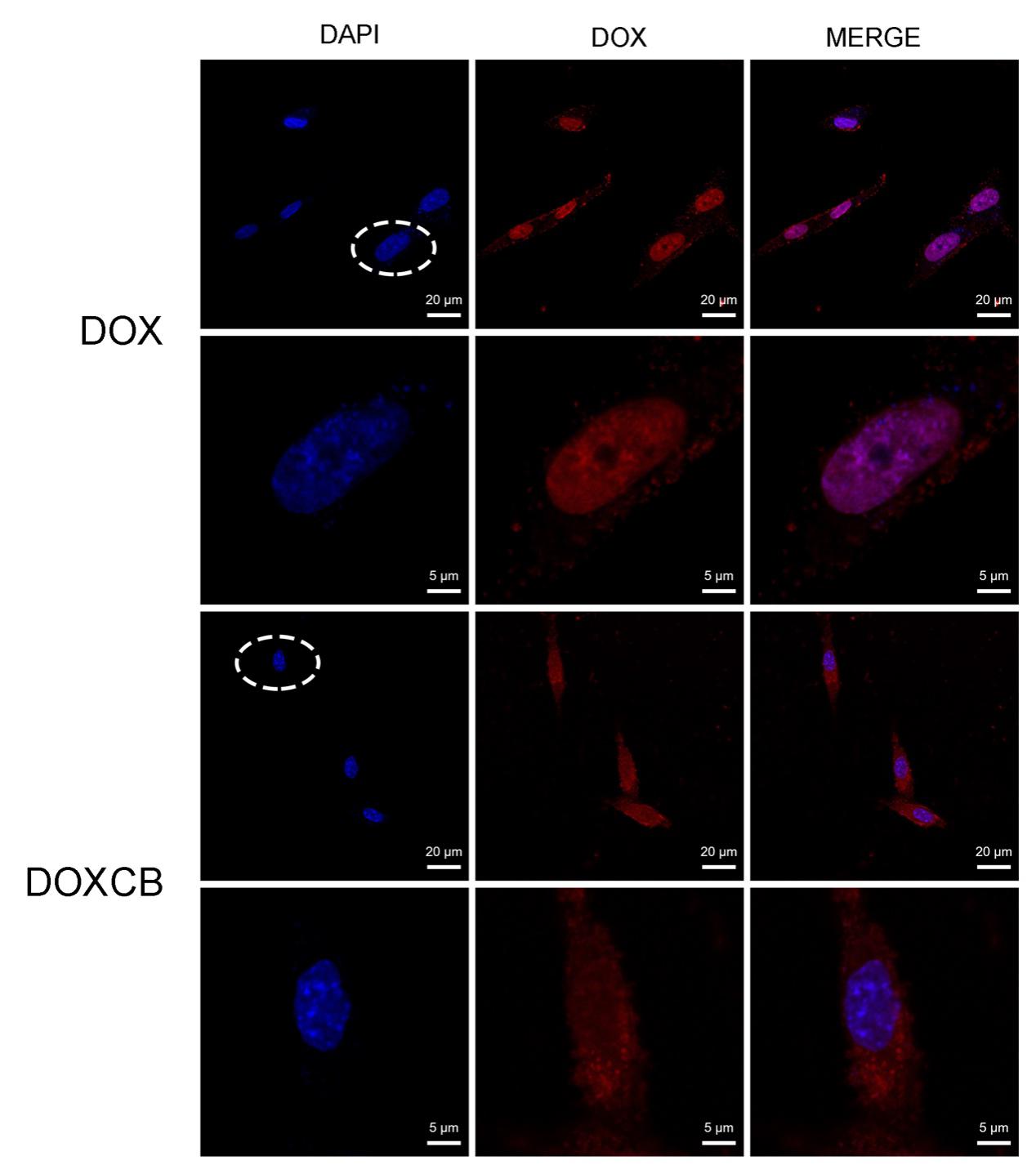


**Figure S11.** Confocal microscopy showing cellular distribution of DOX and DOX-CB. U87 cells were incubated with DOX or DOXCB at the equivalent DOX concentration of 3.5μM for 3h.


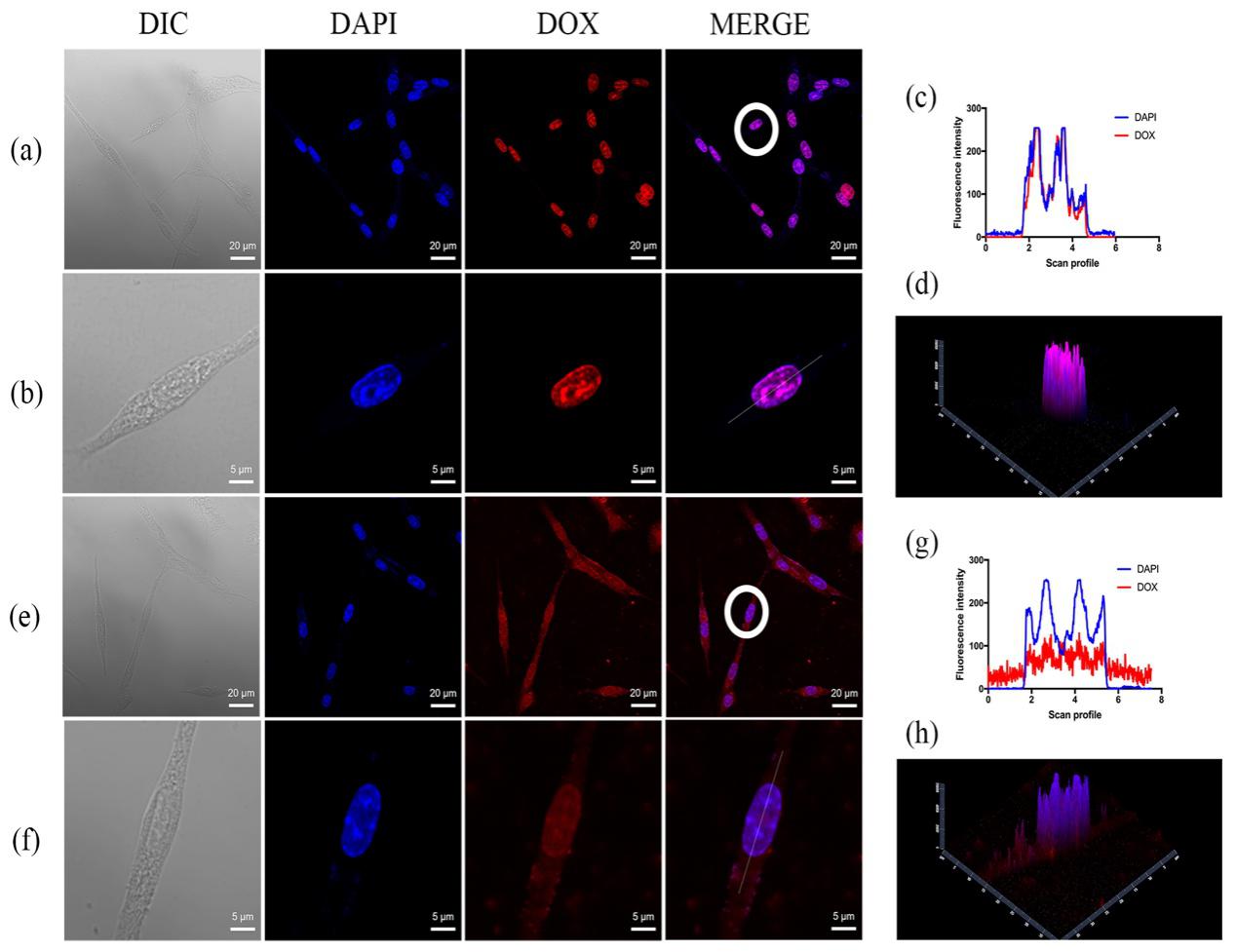


**Figure S12.** Confocal microscopy showing cellular distribution of DOX and DOX-CB. C6 cells were incubated with DOX (a) or DOX-CB (e) at the equivalent DOX concentration of 3.5 μM for 3 h. (b) Themagnification image of selected cell (the white circle region) in (a). (c) Plot scanning profile on selected cell (the white circle region) in (b). (d) 2.5 Dmapping of fluorescence of selected cell (the white circle region) in (b). (f) Themagnification image of selected cell (the white circle region) in (e). (g) Plot scanning profile on selected cell (the white circle region) in (f). (h) 2.5 Dmapping of fluorescence of selected cell (the white circle region) in (f).


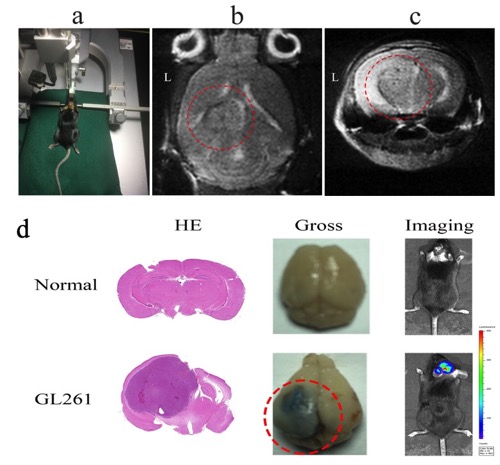


**Figure S13**. GL261-orthotopicglioma in C57BL/6 mouse. (a) Experimental procedure, (b) representative MR images of a cross section in amouse with orthotopic glioma, (c) representative MR images of a coronal section in amouse with orthotopic glioma. (d) HE staining of representative mouse brain sections, gross specimens of brain and in vivo bioluminescent imaging.


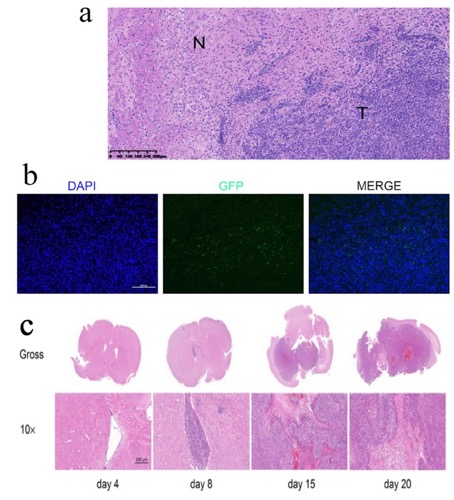


**Figure S14**. Mice bearing established invasive GL261 gliomas. (a) Enlarged HE section of glioma tumor tissue, (b) fluorescent images of GL261-GFP glioma sections. (c) Tumor growth as measured by HE sections of tumors at different time points after GL261 inoculation.


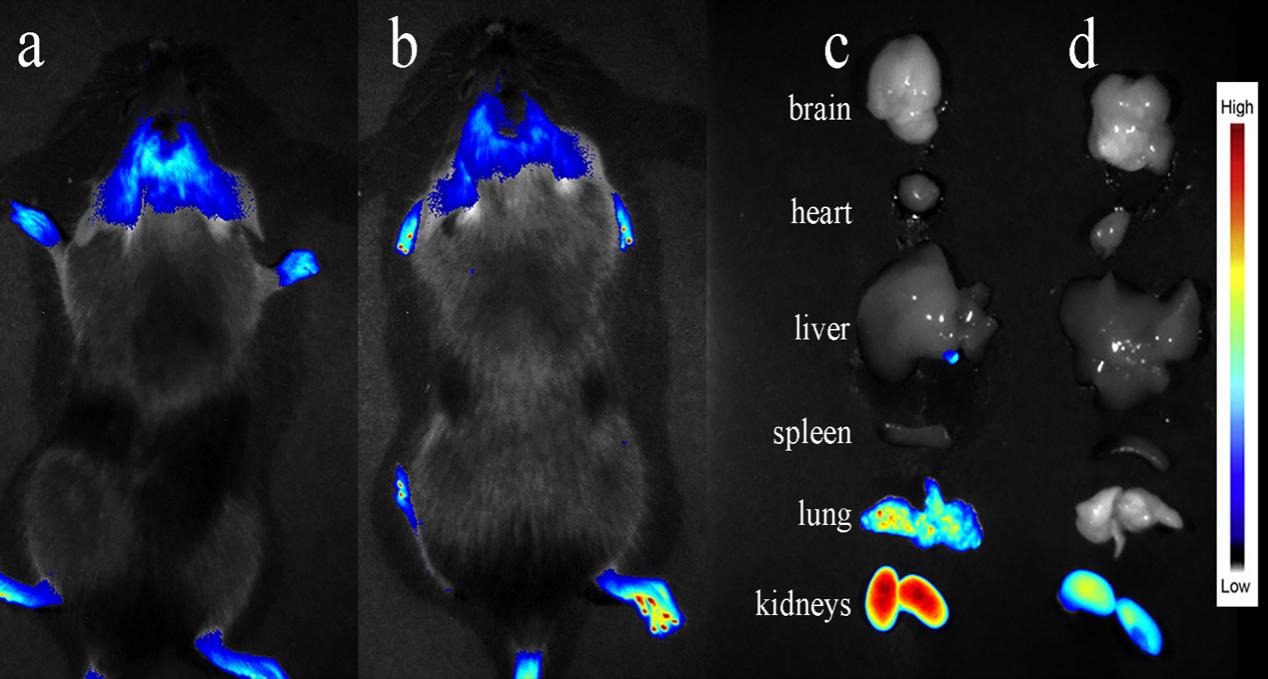


**Figure S15**. *In vivo* and *ex vivo* imaging of C57BL/c mouse bearing GL261-orthotopic glioma 24 h after intravenously injecting DiR (a, c) or liposome encapsulating DiR (b, d). **
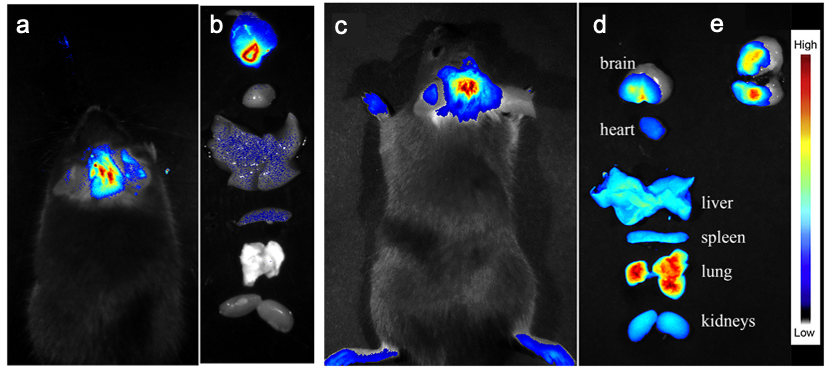
 Figure S16**. *In vivo* (a) and *ex vivo* (b) imaging of C57BL/c mouse bearing GL261-orthotopic glioma 24 h after injecting iRGD-liposome encapsulating DiR in situ. In vivo (c) and ex vivo (d) imaging of C57BL/c mouse bearing GL261-orthotopic glioma 24 h after intravenously injecting iRGD-liposome encapsulating DiR. (e) Fluorescence image of the brain tissue in (c) after coronary resection.


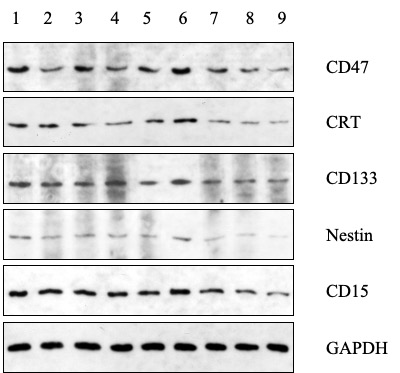


**Figure S17**. Expression of CD47, CRT, Nestin, CD133, CD15 in the GL261 brain tumor tissue harvested from mice administrated with different preparation. Group 1-9 correspond to the DOX-CB@lipo-iRGD-*in situ*-N (+) group , the DOX-CB@lipo-pDNA-iRGD-*in situ*-N (+) group, the DOX-CB@lipo-iRGD-iv-N (+) group, the lipo-pDNA-iRGD-*in situ*-N (+) group, the DOX+CB@lipo-iRGD-iv-N (+) group, the BSH-N (+), group seven: N (+) group, the N (-) group, and the Sham group.


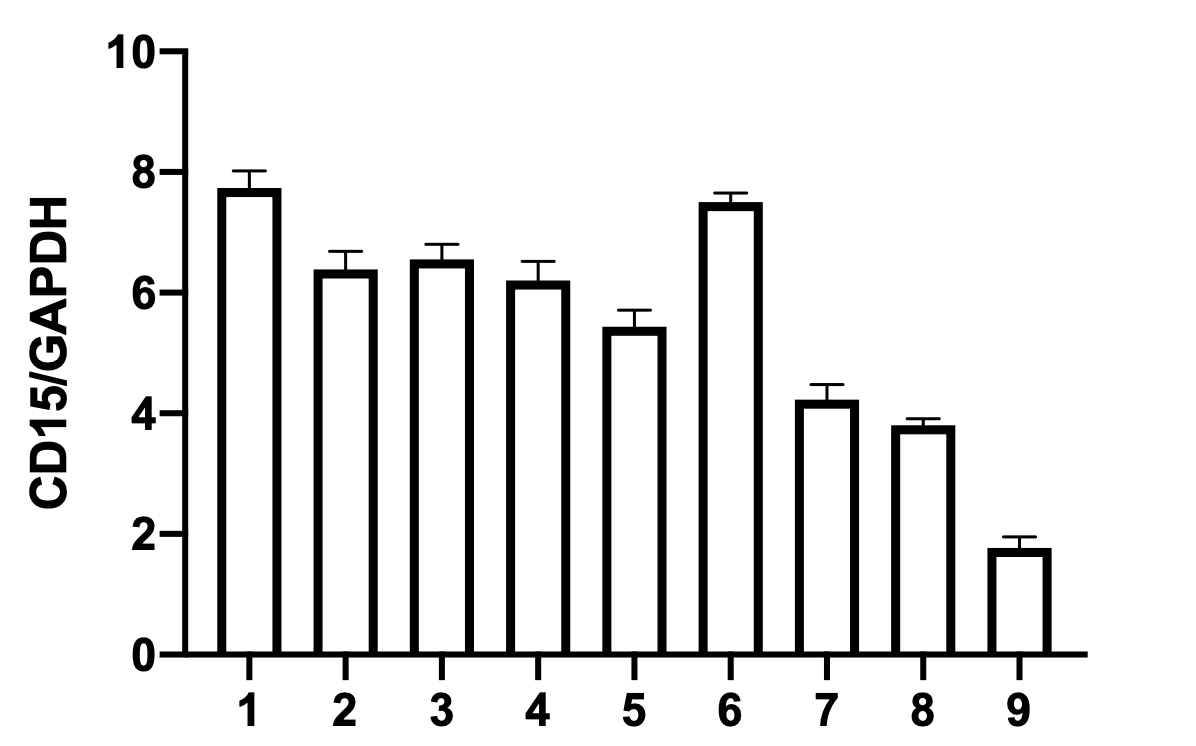


**Figure S18**. Western blot-based quantifications of expression of the CD15 in group 1-9. Group 1-9 correspond to the DOX-CB@lipo-iRGD-*in situ*-N (+) group , the DOX-CB@lipo-pDNA-iRGD-*in situ*-N (+) group, the DOX-CB@lipo-iRGD-iv-N (+) group, the lipo-pDNA-iRGD-*in situ*-N (+) group, the DOX+CB@lipo-iRGD-iv-N (+) group, the BSH-N (+), group seven: N (+) group, the N (-) group, and the Sham group. The results are expressed as mean ± SD (n = 3).


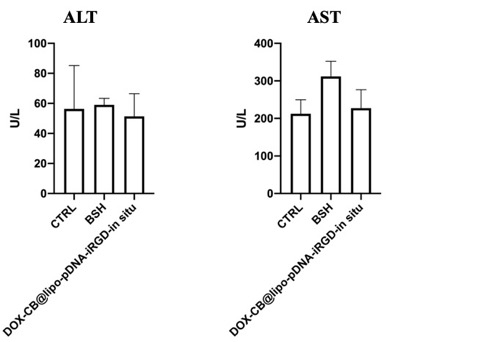


**Figure S19**. Examination of Liver function in control group, BSH group and DOX-CB@lipo-pDNA-iRGD-*in situ* group.


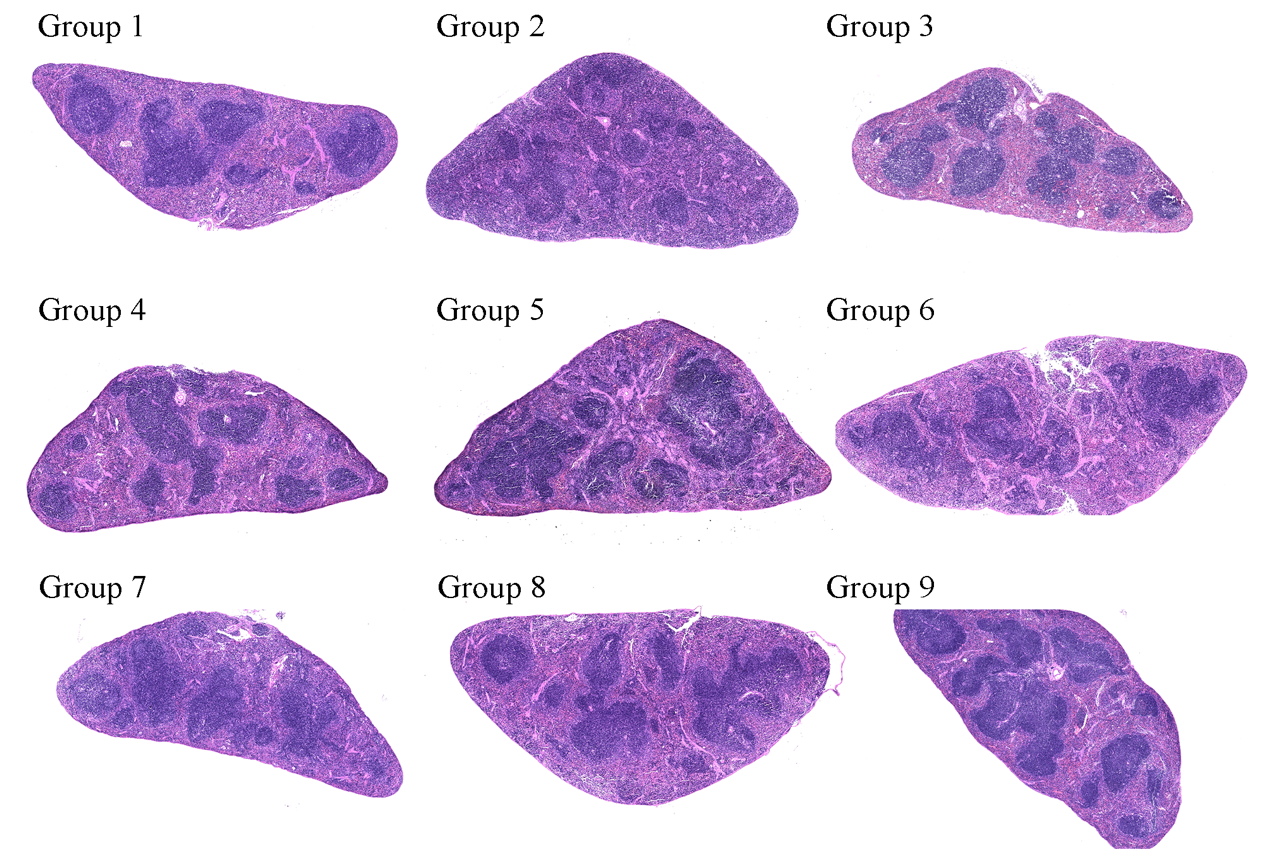


**Figure S20**. Morphology of spleen tissue of mice in Group 1-9. Group 1-9 correspond to the DOX-CB@lipo-iRGD-*in situ*-N (+) group , the DOX-CB@lipo-pDNA-iRGD-*in situ*-N (+) group, the DOX-CB@lipo-iRGD-iv-N (+) group, the lipo-pDNA-iRGD-*in situ*-N (+) group, the DOX+CB@lipo-iRGD-iv-N (+) group, the BSH-N (+), group seven: N (+) group, the N (-) group, and the Sham group.


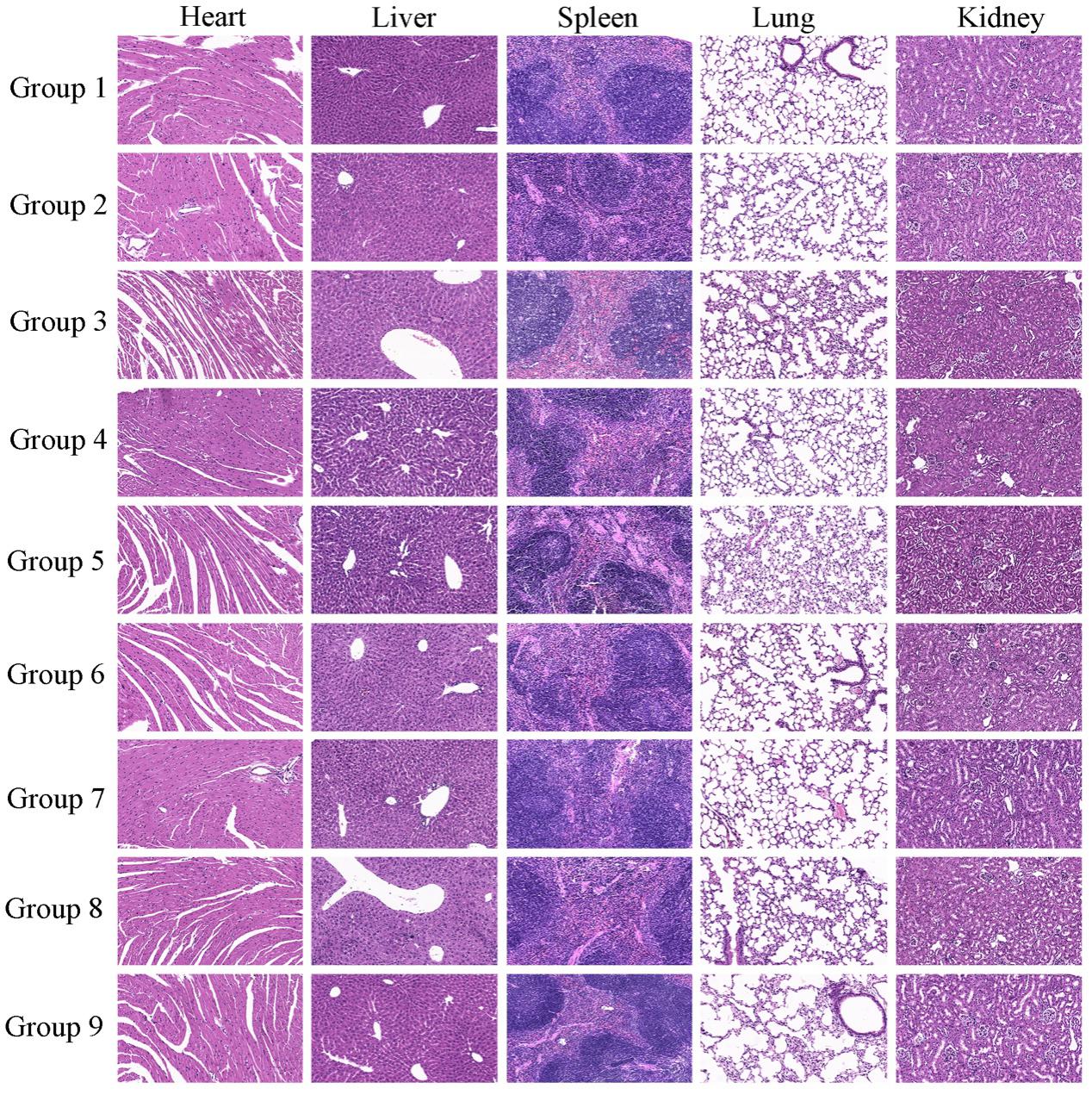


**Figure S21**. HE staining on heart, liver, spleen, lung, kidney in Group 1-9. Group 1-9 correspond to the DOX-CB@lipo-iRGD-*in situ*-N (+) group , the DOX-CB@lipo-pDNA-iRGD-*in situ*-N (+) group, the DOX-CB@lipo-iRGD-iv-N (+) group, the lipo-pDNA-iRGD-*in situ*-N (+) group, the DOX+CB@lipo-iRGD-iv-N (+) group, the BSH-N (+), group seven: N (+) group, the N (-) group, and the Sham group.


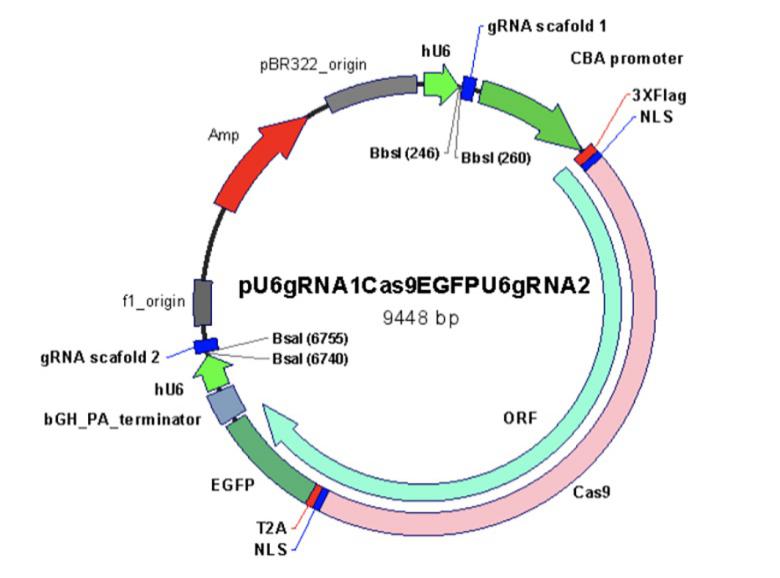


**Figure S22.** Plasmid sequence

**Table S1.** Properties of pDNA-lipoplexes (lipo-pDNA)

| N/P | Size (nm) | PDI | Zeta (mV) |
| --- | --- | --- | --- |
| 0 | 562.97 ± 40.24 | 0.309 ± 0.043 | -25.25 ± 12.63 |
| 1 | 225.25 ± 43.21 | 0.223 ± 0.024 | 2.41 ± 0.82 |
| 2 | 150.35 ± 4.16 | 0.194 ± 0.045 | 12.34 ± 0.51 |
| 3 | 151.48 ± 20.86 | 0.162 ± 0.102 | 18.47 ± 4.78 |
| 4 | 131.61 ± 1.86 | 0.069 ± 0.044 | 16.36 ± 1.08 |
| 5 | 128.64 ± 4.73 | 0.141 ± 0.063 | 18.23 ± 0.30 |
| 10 | 131.84 ± 2.39 | 0.186 ± 0.054 | 23.51 ± 0.28 |
| 20 | 131.10 ± 3.29 | 0.134 ± 0.042 | 25.79 ± 1.45 |
| 30 | 118.98 ± 1.23 | 0.183 ± 0.024 | 22.17 ± 2.78 |
| 40 | 124.60 ± 7.99 | 0.207 ± 0.020 | 25.11 ± 0.77 |
| 50 | 145.21 ± 16.04 | 0.221 ± 0.047 | 24.40 ± 0.97 |
